# Development and preclinical evaluation of next-generation *ΔsigH*-based live candidate vaccines

**DOI:** 10.1172/jci.insight.195947

**Published:** 2025-08-28

**Authors:** Garima Arora, Caden W. Munson, Mushtaq Ahmed, Vinay Shivanna, Annu Devi, Venkata S.R. Devireddy, Basil Antony, Shannan Hall-Ursone, Olga D. Gonzalez, Edward J. Dick, Chinnaswamy Jagannath, Xavier Alvarez, Smriti Mehra, Shabaana A. Khader, Dhiraj K. Singh, Deepak Kaushal

**Affiliations:** 1 Texas Biomedical Research Institute, San Antonio, Texas, USA.; 2 Department of Microbiology, University of Chicago, Chicago, Illinois, USA.; 3 Houston Methodist Research Institute, Houston, Texas, USA.

**Keywords:** Infectious disease, Microbiology, Tuberculosis, Vaccines

## Abstract

To radically diminish tuberculosis (TB) incidence and mortality by 2035, as set out by the WHO End TB Strategy, there is a desperate need for improved TB therapies and a more effective vaccine against the deadly pathogen *Mycobacterium tuberculosis*. Aerosol vaccination with the *MtbΔsigH* mutant protects 2 species of nonhuman primates against lethal TB challenge by invoking vastly superior T and B cell responses in the lungs through superior antigen presentation and interferon conditioning. Since the Geneva Consensus on essential steps toward the development of live mycobacterial vaccines recommends that live TB vaccines incorporate at least 2 independent gene knockouts, we have now generated several rationally designed, double-knockout (DKO) and triple-knockout (TKO) mutants in *Mtb*, each containing the *ΔsigH* deletion. Here, we report preclinical studies in the rhesus macaque model of aerosol infection and SIV/HIV coinfection, aimed at assessing the safety of these *MtbΔsigH*-based DKOs and TKOs. We found that most of these mutant strains were attenuated in both immunocompetent and SIV-coinfected macaques, and combinatorial infection with these generated strong cellular immune responses in the lung, akin to *MtbΔsigH*. Aerosol infection with these KO strains elicited inducible bronchus-associated lymphoid tissue, which is a correlate of protection from TB.

## Abstract 2

New, efficacious vaccines are needed to control TB globally. We have shown at a non-disease causing lab strain of TB can protect monkeys against TB. However, elaborate guidelines exist for the development of such vaccines. We have now generated strains of TB which comply with these guidelines. We show that these strains are safe and immunogenic in both immune-competent and -compromised monkeys, paving the way for the testing of these new vaccine candidates.

## Introduction

Tuberculosis (TB) is a worldwide health crisis, claiming 1.6 million lives annually, infecting over 10 million people, and placing severe financial burdens on patients while driving up health care costs (1). New TB vaccines are among the most effective tools for curbing the spread of drug-resistant TB, decreasing reliance on antibiotics, limiting the emergence of drug resistance, and disrupting transmission (2–5). Studies indicate that TB vaccines have the potential to save millions of lives and reduce the economic burden associated with TB-related drug resistance (6). The widely used BCG vaccine is only partially protective in children and adults (7, 8).

Live-attenuated *Mycobacterium tuberculosis* (*Mtb*) represent a class of TB vaccine candidates that can potentially elicit highly protective and durable responses, as these candidates closely mimic the antigenic repertoire of the pathogen (9–11). However, there can be concerns about the safety of such vaccine candidates (12). As a result, only one such live-attenuated *Mtb* vaccine (MTBVAC) is in advanced clinical trials (13), and very few are in preclinical development. The aim of this study is to develop a breakthrough human TB vaccine candidate that could eventually proceed to clinical development. Previous studies have shown that the *Mtb* isogenic mutant in *sigH* (*ΔsigH*) is attenuated for replication and disease in macaques (14–16). This allele encodes SigH, an alternative sigma factor of *Mtb* that is activated by heat, nitric oxide and oxidative stress, acidic pH, enduring hypoxia, and phagocytosis (17–22). It activates the thioredoxin/thioredoxin reductase system, antioxidants that neutralize peroxides and maintain redox balance, enabling the detoxification of key host defense mechanisms (17).

Aerosol vaccination with *MtbΔsigH* induces strong local immune responses that protect macaques against lethal TB (15, 16). These include robust B cell responses resulting in the formation of significant inducible bronchus-associated lymphoid tissue (iBALT) in both rhesus (15) and cynomolgus (16) macaques. When B cells were depleted, the ability of *ΔsigH* to protect macaques from a lethal TB challenge was reduced (23). In the more resistant cynomolgus model, aerosol vaccination with *ΔsigH* induced high levels of type I interferon (IFN), resulting in superior T cell priming, without causing any pathological effects typically associated with type I IFN signaling (16). This was associated with elite protection of *ΔsigH*-vaccinated macaques from TB disease (16). Furthermore, nonpathogenic *ΔsigH* infection in rhesus macaques is not reactivated by SIV coinfection (24).

Although *ΔsigH* has shown strong immune protection, safety, immunogenicity, and efficacy in various macaque models, safety concerns may arise regarding the use of this candidate vaccine in humans, as it only contains a single gene deletion in *Mtb*. To meet the Geneva Consensus for developing live attenuated *Mtb* vaccines, additional unrelated mutations must be incorporated into *ΔsigH* to facilitate its clinical development (25). We have created 8 rationally attenuated vaccine candidates, including double- and triple-gene knockout strains derived from *Mtb* CDC1551 *ΔsigH*, such as *MtbΔsigHΔsecA2* and *MtbΔsigHΔfbpAΔsapM*. While some of these mutants generate immune enhancement- or auxotrophy-based attenuation phenotypes, others render *Mtb* avirulent in macaque lungs (26). We hypothesized that the addition of *ΔsigH* would render these mutants more immunogenic, while decreasing persistence, thus enhancing safety. Here, we assessed the safety and immunogenicity of these multiple knockout strains of *Mtb* (*Mtb* KOs) at a high dose in combination in rhesus macaques. We also evaluated the safety of these strains via coinfection with SIV. We hypothesized that if these *Mtb* KOs were not sufficiently attenuated, the macaques would develop signs of TB disease, such as granulomatous pathology and uncontrolled bacterial replication in the lungs, similar to what has been observed with BCG in this model (27).

## Results

### Identifying genes to be deleted in MtbΔsigH.

Throughout its life cycle in the host, *Mtb* encounters many stress conditions, the response to which is regulated by SigH, playing a key role in maintaining its viability (17–22). The *ΔsigH* mutant is attenuated ex vivo (16, 28), as well as in macaques (14), where it induces strong lung immune signatures that protect against lethal TB (15, 16). Although SIV coinfection does not reactivate infection with the *MtbΔsigH* in macaques (24), additional unrelated mutations are required to ensure its clinical safety (25, 29). To develop *MtbΔsigH*-based TB vaccine candidates that meet the Geneva Consensus recommendations for safe live TB vaccines with strong potential for clinical advancement (25, 29), we selected genes, deletion in which attenuated the ability of *Mtb* to colonize macaque lungs. To achieve this, we identified 8 specific genes (Table 1) to be deleted in *ΔsigH*. Building on these previous studies, the selected genes and strains were (i) *ΔfbpAΔsapM*, (ii) *ΔsecA2ΔsodA*, (iii) *ΔleuD* (21–23), (iv) *ΔmetA* (24), (v) *Δmce4EΔmce4F*, (vi) *ΔhadC*, (vii) *ΔMT3785*, and (viii) *Δmce1A*. The proteins encoded by the *Mtb* genes, *fbpA* and *sapM*, interfere with phagolysosomal maturation in host, and therefore, the *Mtb* *ΔfbpAΔsapM* strain has been shown to be significantly attenuated in murine and human macrophages due to increased phagolysosomal fusion that facilitates an increased presentation of Ag85B to CD4^+^ T cells (30–35). This mutant is immunogenic and protective in macrophages and mice (30). Similarly, SecA2 facilitates the arrest of phagolysosomal fusion, enhancing *Mtb* survival in host and transports proteins involved in pathogenesis, such as SodA, SapM, and PknG (36–38). As expected, *Mtb* *ΔsecA2Δsod*A is highly attenuated and exhibits enhanced antigen presentation and phagolysosomal fusion capability (39). The *MtbΔsecA2* mutant is also efficacious as a TB vaccine and safe in SIV-coinfected macaques (40). *Mtb* *ΔleuD* (41) and *ΔmetA* (42) are attenuated auxotrophs. *ΔleuDΔpanCD* (43) and *ΔmetA* strains of *Mtb* are safe in SCID mice, guinea pigs, and immunocompetent and SIV-coinfected macaques and exhibit protection against *Mtb* challenge (43–46). The remaining genes were selected because transposon interruption in these alleles rendered *Mtb* avirulent in macaques and in various other screening studies involving in silico, macrophage, or murine models (26, 47–51). None of the products encoded by these genes are related to *sigH* function or signaling. Deletion mutants of several of these genes have been individually shown to be attenuated due to dysregulation in cholesterol (*mce4*) or mycolic acid (*mce1*) transport (52–54).

### Construction and in vitro characterization of ΔsigH-based double- and triple-knockout strains of M. tuberculosis.

**Figure 1:**
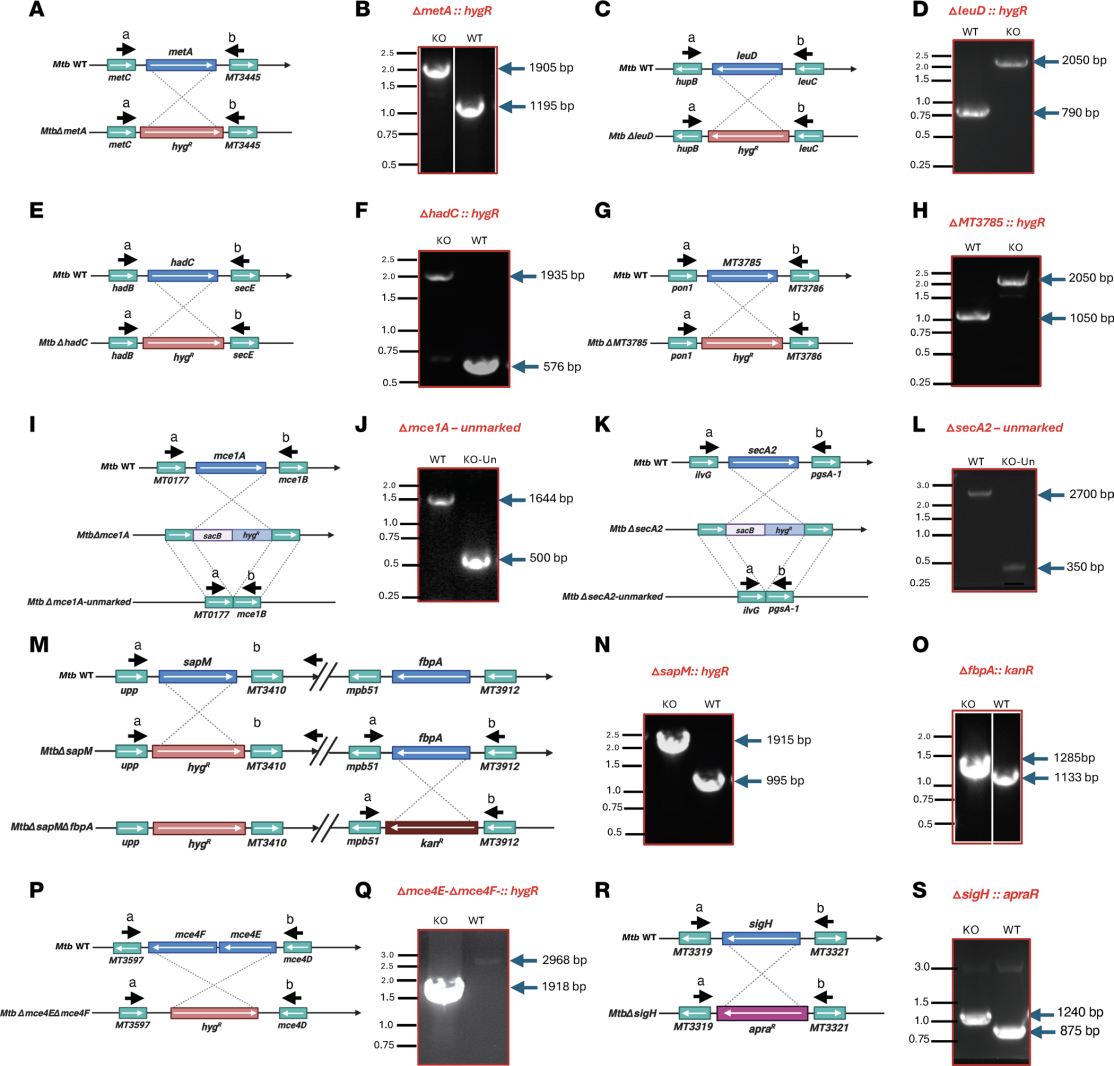
Construction of various double- and triple-knockout strains of *M*. *tuberculosis*. Schematic representation of gene locus and PCR-based analysis in the parental (wild-type) and deletion strains of *M*. *tuberculosis* CDC1551 are shown. Created in BioRender. Arora, G. (2025) https://BioRender.com/qa76loa The open reading frames of *metA* (**A** and **B**), *leuD* (**C** and **D**), *hadC* (**E** and **F**), *MT3785* (**G** and **H**), *mce4E-mce4F* (**P** and **Q**), *mce1A* (**I** and **J**), *secA2* (**K** and **L**), and *sapM* (**M** and **N**) were separately replaced with the hygromycin resistance gene (*hyg^R^*) in the *M*. *tuberculosis* genome. *MtbΔmce1A* and *MtbΔsecA2* were also unmarked in another phage transduction step using the temperature-sensitive mycobacteriophage phAE280 (unmarking phage; ref. 56). In the double-mutant strain, *MtbΔsapMΔfbpA*, the open reading frame of *fbpA* was replaced with kanamycin resistance gene (*kan^R^*) in the genome of the *MtbΔsapM* strain (**M** and **O**). *sigH* was replaced with apramycin resistance gene (*apra^R^*) in each of the 8 mutant strains. (**R** and **S**) The disruptions of various genes, in their respective single- and double-mutant strain, were confirmed by PCR amplification using locus-specific primers. The solid black arrows depict the region of binding by the primers for PCR-based screening. The lanes presented in each panel are derived from the same gel, with images cropped for clarity. Source data are provided as a Supporting Data Values file.

We first constructed the isogenic single mutants, *ΔsapM::hyg^R^*, *Δmce1A::hyg^R^*, *ΔsecA2*::*hyg^R^*, *ΔhadC::hyg^R^*, *ΔMT3785::hyg^R^*, *ΔmetA::hyg^R^*, *ΔleuD::hyg^R^*, and *ΔfbpA::kan^R^* as well as the double mutant, *Δmce4E/Δmce4F::hyg^R^*, in *Mtb* CDC1551 (Figure 1) using a specialized transducing phage-based system (55). The entire open reading frame of each gene was replaced by either a hygromycin (*hyg^R^*) or kanamycin (*kan^R^*) resistance gene via homologous recombination using the fragments immediately up- and downstream of the coding sequence (Figure 1). The deletions of *secA2* and *mce1A* involved the removal of *hyg^R^* selectable markers using γδ resolvase via phage transduction (56), resulting in unmarked mutants, *MtbΔsecA2*-un and *MtbΔmce1A*-un (Figure 1, I–L). The *sigH* deletion was attained in the background of these single and double mutants by transduction with the recombinant phage phAE87-*ΔsigH::apra^R^*, a phagemid DNA carrying the upstream and downstream regions flanking *sigH* on either side of an apramycin resistance (*apra^R^*) cassette in a cosmid, pYUB854. We were then able to delete *sigH* by replacing the gene with an *apra^R^* cassette in each individually generated knockout strain of *Mtb* (Figure 1, R and S). The deletions and replacements were confirmed by PCR analysis using either the external primer set flanking the gene or internal locus-specific primers or a mixture of both (Figure 1). The results were also verified by sequencing (data not shown). These observations confirmed that the open reading frames for the genes, *sapM*, *fbpA*, *mce4E*, *mce4F*, *mce1A*, *secA2*, *hadC*, *MT3785*, *metA* and *leuD*, had been individually deleted in the *MtbΔsigH* strain.

**Figure 2:**
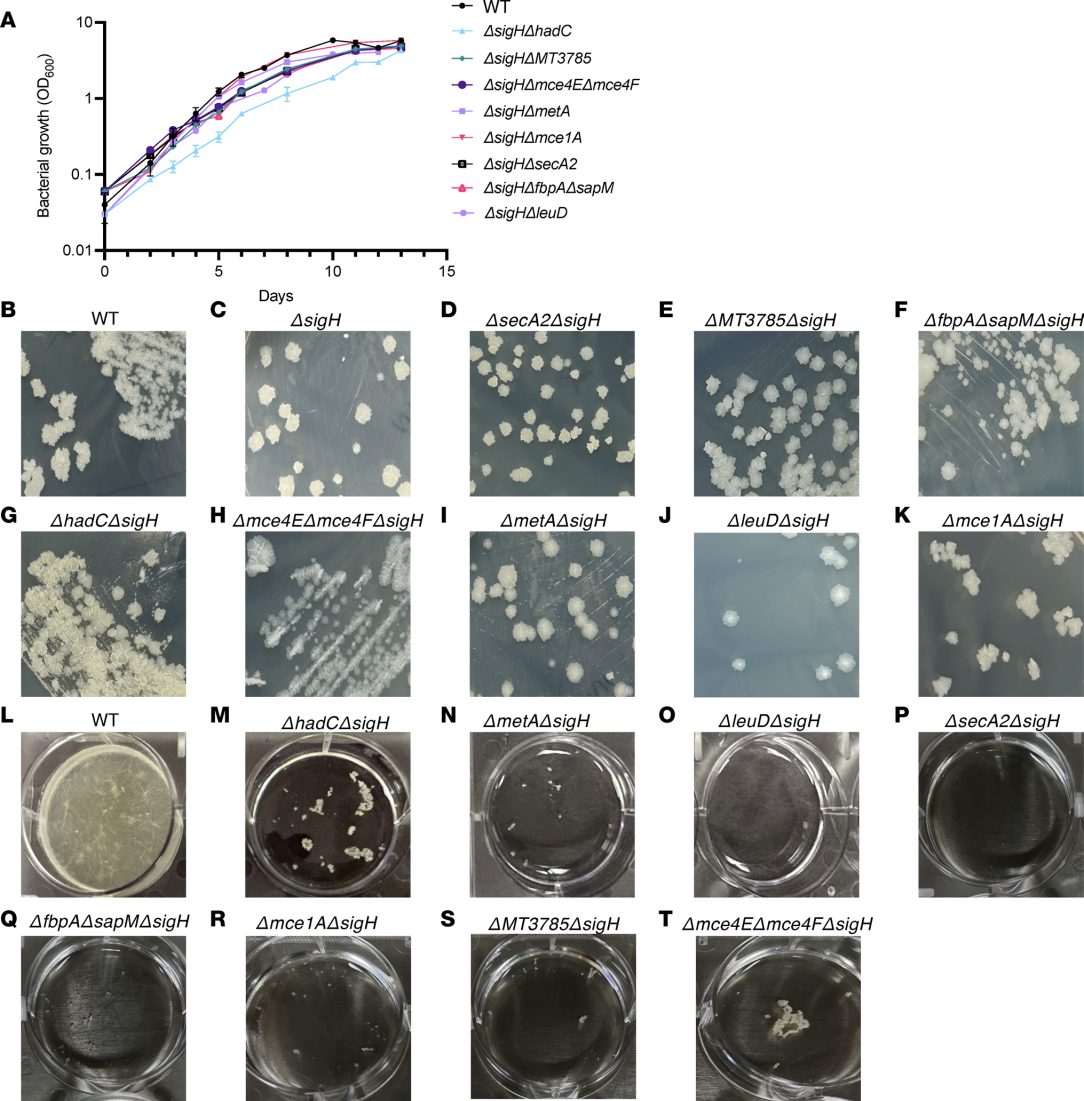
In vitro characterization of *MtbΔsigH*-based KO strains. (**A**) Log-phase cultures of individual knockout or wild-type *M*. *tuberculosis* CDC1551 grown in standard MB7H9 medium were diluted 1/100, and growth kinetics at 37°C was followed by measuring optical density at 600 nm (OD_600_) over time. Colony morphology of various *Mtb* KO strains was determined by cultivating 10-fold serial dilutions of the early- to mid-log phase cultures on standard MB7H11 plates at 37°C for 3–4 weeks (**B**–**K**). Loss of biofilm formation in *MtbΔsigH*-based KO strains depicted by growing various strains in Sauton’s medium in polystyrene-coated, 6-well plates without shaking at 37°C (**L**–**T**). The plates were imaged after 4–5 weeks of incubation. The data shown in all the panels are representative of 2 independent experiments.

As an initial step in assessing the contribution of each gene to bacterial fitness, we monitored the growth of the mutants in liquid cultures. As shown in Figure 2A, the growth curves of the double (*MtbΔsigHΔsecA2*, *MtbΔsigHΔmce1A*, and *MtbΔsigHΔMT3785*) and triple knockouts (*MtbΔsigHΔfbpAΔsapM* and *MtbΔsigHΔmce4EΔmce4F*) were comparable to parental *Mtb*, with each strain reaching a similar plateau value with similar growth rates. *MtbΔsigHΔmetA* and *MtbΔsigHΔleuD* also exhibited growth patterns similar to the wild-type upon supplementation with methionine and leucine, respectively, consistent with previous findings for these auxotrophic mutants of *Mtb* (41, 42). *MtbΔsigHΔhadC* grew at a slightly slower rate compared with wild-type and other strains (Figure 2A). The colony morphology of all the mutant strains was also comparable to that of wild-type *Mtb* (Figure 2, B–K). Each strain produced dry rough colonies characterized by ridges and well-defined borders (Figure 2, B–K). As reported earlier for *MtbΔhadC*, compared with wild-type and all other strains, *MtbΔsigHΔhadC* strain exhibited a revival-defective phenotype and a longer lag phase (57). In addition, the colony morphology of *MtbΔsigHΔhadC* was noticeably different, with changes in texture and diminished pigmentation relative to the wild-type, consistent with the previous findings for the inactivation of *hadC* in *Mtb* genome (Figure 2G) (58). Biofilm formation by *Mtb* facilitates its survival within the host and confers increased drug tolerance while hindering immune cell activity and evading host defenses (59–61). Strikingly, we found that all mutant strains were severely compromised in their ability to form biofilm in vitro (Figure 2, L–T), including the isogenic mutant for *MtbΔsigH*. To test whether the mutations introduced altered the drug susceptibility of parental strain, we measured the minimum inhibitory concentrations (MICs) of *Mtb* KOs strains to most frequently used first- and one second-line antitubercular drugs. As shown in Supplemental Table 1 (supplemental material available online with this article; https://doi.org/10.1172/jci.insight.195947DS1), all the mutant strains were sensitive to rifampin (RIF), isoniazid (INH), and ethambutol (ETH). The MIC_99_ values of RIF against the mutant strains were <4 μM. The values fell between 0.39 μM and 0.78 μM for INH and were 1.56–3.12 μM for ETH (Supplemental Table 1).

### Challenge with MtbΔsigH-based KOs does not cause TB disease.

**Figure 3:**
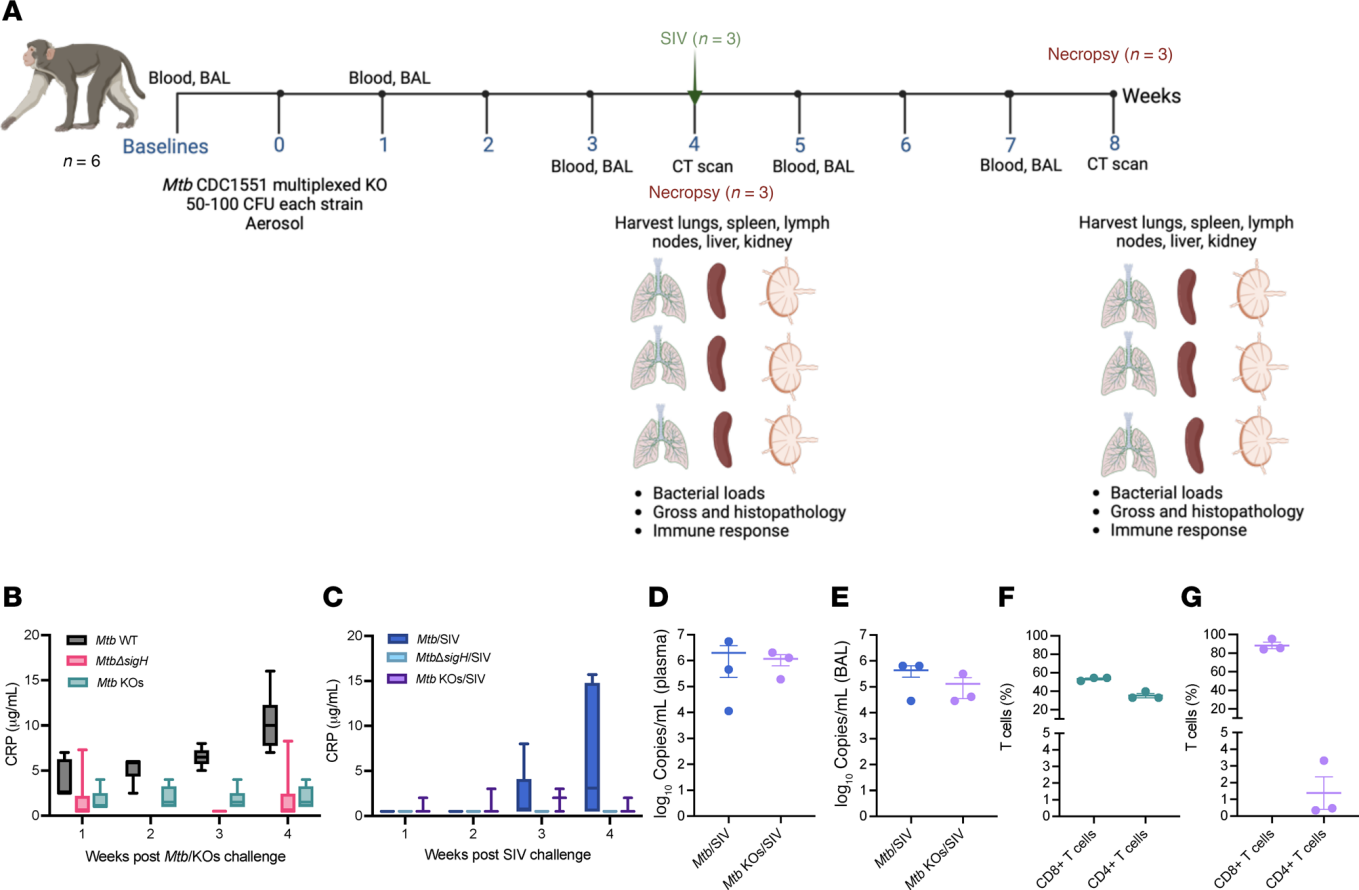
Clinical outcomes of *Mtb* KOs and subsequent SIV challenge in rhesus macaques. (**A**) Study outline. Created in BioRender. Kaushal, D. (2025) https://BioRender.com/q65y137 Six Indian rhesus macaques were challenged with a mixture of all 8 *MtbΔsigH*-based knockouts with a targeted dose of 50–100 CFU of each strain. Shown are serum C-reactive protein (CRP) levels after *Mtb* KOs challenge (**B**), serum CRP levels after SIV challenge (**C**), and viral loads in plasma (**D**) and BAL supernatants (**E**) of the macaques measured at 4 weeks after SIV challenge. CD4^+^ and CD8^+^ T cells were measured in BAL at 4 weeks after *Mtb* KOs challenge (**F**) and 4 weeks after SIV challenge (**G**). Control data shown (for *Mtb* wild-type, *MtbΔsigH*, *Mtb*/SIV, and *MtbΔsigH*/SIV) for comparison were obtained from our previously published studies. Box plots show the interquartile range, median (line), and minimum and maximum (whiskers) (**B** and **C**). Data are represented as mean ± SEM (**D**–**G**).

*MtbΔsigH*, when aerosolized, is safe and provides better protection in macaques against lethal TB than BCG in various nonhuman primate models (14–16). We investigated if the additional mutations also protected the host while remaining nonpathogenic. To test the safety of various knockout strains, as outlined in Figure 3A, we aerosol-challenged 6 macaques with a pool of 50–100 CFU of each knockout strain. Three of the 6 animals were euthanized at 4 weeks postchallenge to assess bacterial burden and disease pathology, while the remaining 3 nonhuman primates (NHPs) were given a 4-week rest period before being challenged intravenously with a high dose of SIVmac239 (Figure 3A). Infection and disease were monitored through weekly clinical assessments and serial bronchoalveolar lavage (BAL) sampling. For comparison, control data shown in the graphs include *Mtb* CDC1551–latently infected rhesus macaques, which were further challenged with SIV at 9 weeks postinfection (*Mtb*/SIV), as well as those aerosol-vaccinated with 1,000 CFU of *MtbΔsigH* (24) (Figure 3, B and C, and Supplemental Figure 1).

Following the *Mtb* KOs challenge as well as subsequent high-dose intravenous SIV infection, all animals remained healthy and devoid of TB disease. All *Mtb* KOs-infected and *Mtb* KOs/SIV-coinfected NHPs either maintained or gained body weights and exhibited stable body temperatures compared with single *MtbΔsigH* mutant-infected and *Mtb*/SIV-coinfected animals, as shown by the change in these parameters relative to baseline values (Supplemental Figure 1). All *Mtb* KOs-infected and SIV-coinfected animals maintained normal CRP values throughout the study, indicating the absence of marked inflammation or disease, whereas CRP levels in the control groups (*Mtb*- and *Mtb*/SIV-challenged) increased over time (Figure 3, B and C). SIV titers measured in plasma and BAL supernatants were comparable between the control and test groups, indicating that the observed differences were not due to variations in viral loads (Figure 3, D and E). The serum albumin/globulin ratios were elevated in the *Mtb* KOs-challenged animals, suggesting reduced TB disease severity (Supplemental Figure 2A). The neutrophil/lymphocyte ratios in peripheral blood of *Mtb* KOs-infected animals were lower relative to *Mtb*/SIV-challenged animals, further indicating the healthy status of the *Mtb* KOs-challenged animals (Supplemental Figure 2B). At 4 weeks after *Mtb* KOs challenge, BAL demonstrated a considerably high percentage (30%–50%) of CD4^+^ T and CD8^+^ T cells (Figure 3F). As expected, at week 8 after *Mtb* KOs challenge/week 4 after SIV challenge, substantial CD4^+^ depletion was observed (Figure 3G).

### MtbΔsigH-based KOs challenge reduces in vivo bacterial burdens and does not disseminate to extrathoracic organs.

**Figure 4:**
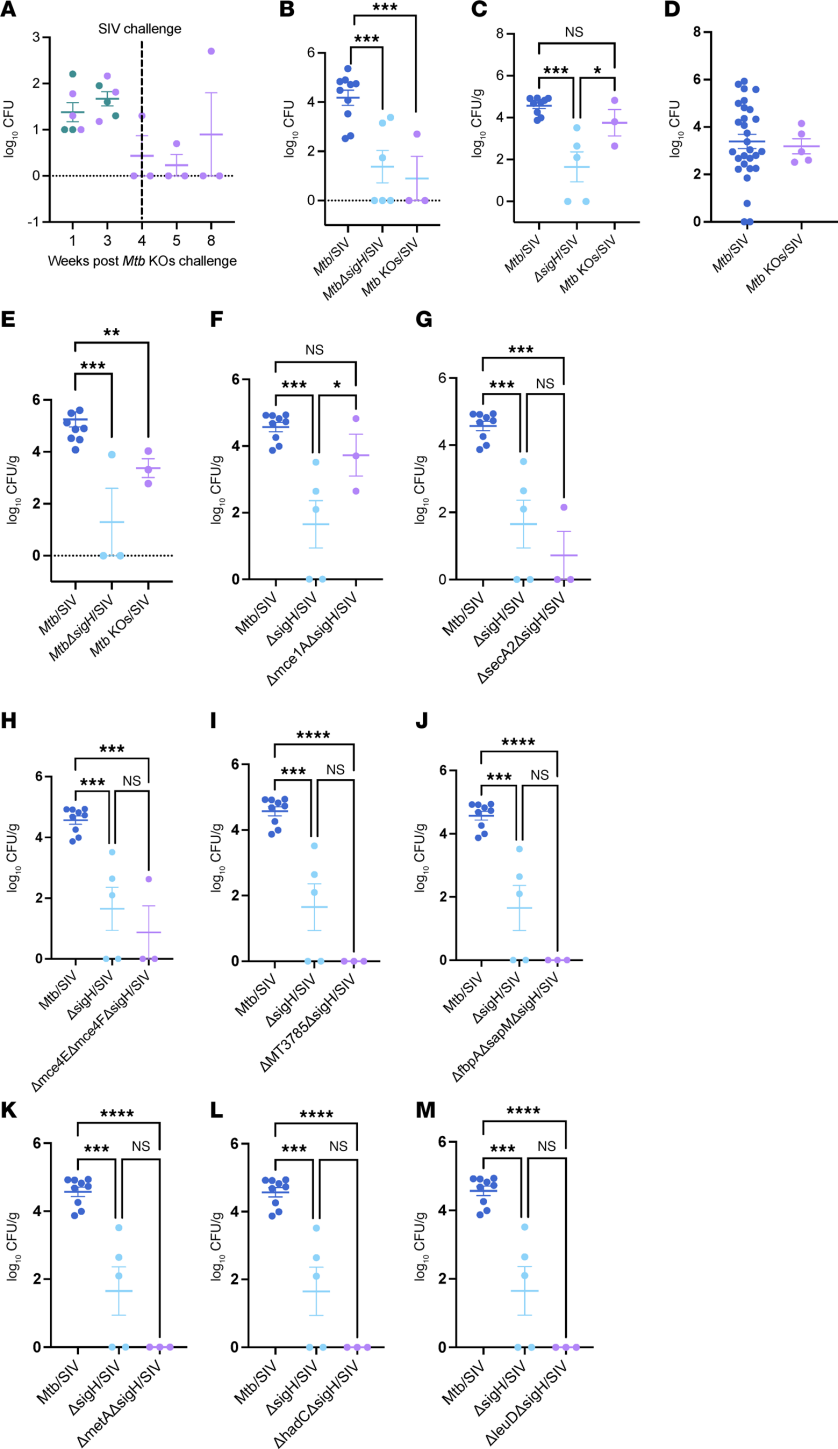
Thoracic bacterial burden in rhesus macaques following *Mtb* KOs or *Mtb* KOs/SIV coinfection. Rhesus macaques challenged with *Mtb* KOs strains via aerosol route and subsequently with SIV intravenously were euthanized at the indicated time points and analyzed for bacterial burdens. BAL CFU were measured longitudinally throughout the study (**A**). The dashed line indicates the time of SIV challenge. Bacillary loads recorded in endpoint BAL (**B**), lung (**C**), lung granulomas (**D**), and BrLN (**E**) from *Mtb* KOs/SIV (lavender) challenged animals were compared with the *Mtb*/SIV (blue) and *MtbΔsigH*/SIV (turquoise) infected animals from earlier studies. (**F**–**M**) Strain-specific bacterial burden determined by PCR analysis of lysates from colonies isolated from BAL fluid, lung tissue, and BrLN collected at necropsy. Data involving *Mtb*/SIV and *MtbΔsigH*/SIV-coinfected macaques are obtained from our previous publications (23, 82, 83), as we did not perform experiments on this group in the current study. The data were analyzed using 1-way ANOVA with Tukey’s multiple comparisons test in GraphPad Prism version 9.2.0 for macOS. A *P* value < 0.05 was considered as statistically significant. *P < 0.05; **P < 0.01; ***P < 0.001; ****P < 0.0001. Data are presented as mean ± SEM.

The primary indicator of protection is the thorough quantification of *Mtb* burden at necropsy. CFU in the BAL of animals decreased by 75% after 3 weeks of challenge and remained low even after the SIV challenge (Figure 4A). As shown in Figure 4, A and B, 1 out of 3 SIV-challenged macaques had higher bacterial burden at the time of necropsy but remained free of the disease as indicated by clinical parameters. As shown in Figure 4, C and E, the lung and bronchial lymph node (BrLN) bacillary loads in *Mtb* KOs/SIV-infected animals were 3.8- and 3.4-log_10_, respectively, in comparison with *Mtb*/SIV-infected animals, where these were 4.6- and 5.2-log_10_. Despite the high bacterial burdens, we observed 6.0-fold fewer granulomas in lungs of *Mtb* KOs/SIV-infected animals compared with wild-type/SIV control-infected NHPs (Figure 4D). *Mtb*/HIV coinfection in humans is marked by widespread dissemination to extrathoracic organs. Therefore, we next evaluated the bacterial loads in spleen, liver, and kidneys. *Mtb*/SIV–coinfected macaques exhibited significant extrapulmonary dissemination as evident by high bacterial burdens (2–4 log_10_ CFU) in spleen, liver, and kidneys (Supplemental Figure 3). In contrast, no bacilli were detected in any of the collected extrapulmonary tissues of *Mtb* KOs/SIV-infected macaques (Supplemental Figure 3). These results demonstrate *MtbΔsigH*-based KOs-challenged macaques remain asymptomatic of tuberculous disease throughout the study despite active replication in the lung and when infected with a high dose of pathogenic SIV did not experience reactivation of TB disease.

### MtbΔsigHΔmce1A: the predominant replicating strain in the Mtb KOs-challenged macaques.

The *Mtb* *mce1* operon has been implicated in fatty acid uptake (52, 62). Previous studies have shown that the *Δmce1A* mutant of *Mtb* Erdman exhibits increased growth in BALB/c mice, leading to earlier mortality compared with mice infected with the parental strain (63). We hypothesized that the majority of the total thoracic bacterial burden in our animals could comprise the *MtbΔsigHΔmce1A* strain, given its known hypervirulent phenotype in mice. We performed PCR analysis on lysates prepared from colonies isolated from the BAL, lungs, and BrLNs at necropsy. This analysis was expected to reveal an amplification size corresponding to the *Δmce1A* genotype. Approximately 20% of the total CFU recovered from all animals were screened, and as anticipated, mutations were mapped to *mce1A* in 91% of the colonies obtained from lungs (Supplemental Figure 4). PCR analysis of the remaining 9% of the total screened clones identified them as either *MtbΔsigHΔsecA2* or *MtbΔsigHΔmce4EΔmce4F* mutants (data not shown). Thus, *MtbΔsigHΔmce1A* strain accounted for 3.7-log_10_ CFU, while *MtbΔsigHΔsecA2* and *MtbΔsigHΔmce4EΔmce4F* strains contributed 0.7-log_10_ CFU and 0.9-log_10_, respectively, to the total lung bacterial load (Figure 4, F–H). The remaining 5 mutant strains did not replicate in the lungs (Figure 4, I–M). None of the *Mtb* KOs other than *ΔsigHΔmce1A* survived in BAL and BrLN, as all the colonies recovered from these tissues carry the *mce1A* deletion (Supplemental Figure 4). These results suggest that the *mce1A* deletion likely enabled this strain to dominate and replicate in the thoracic region, while the *sigH* deletion continued to provide protection to the animals.

### Challenge with MtbΔsigH-based KOs leads to reduced pulmonary pathology.

**Figure 5:**
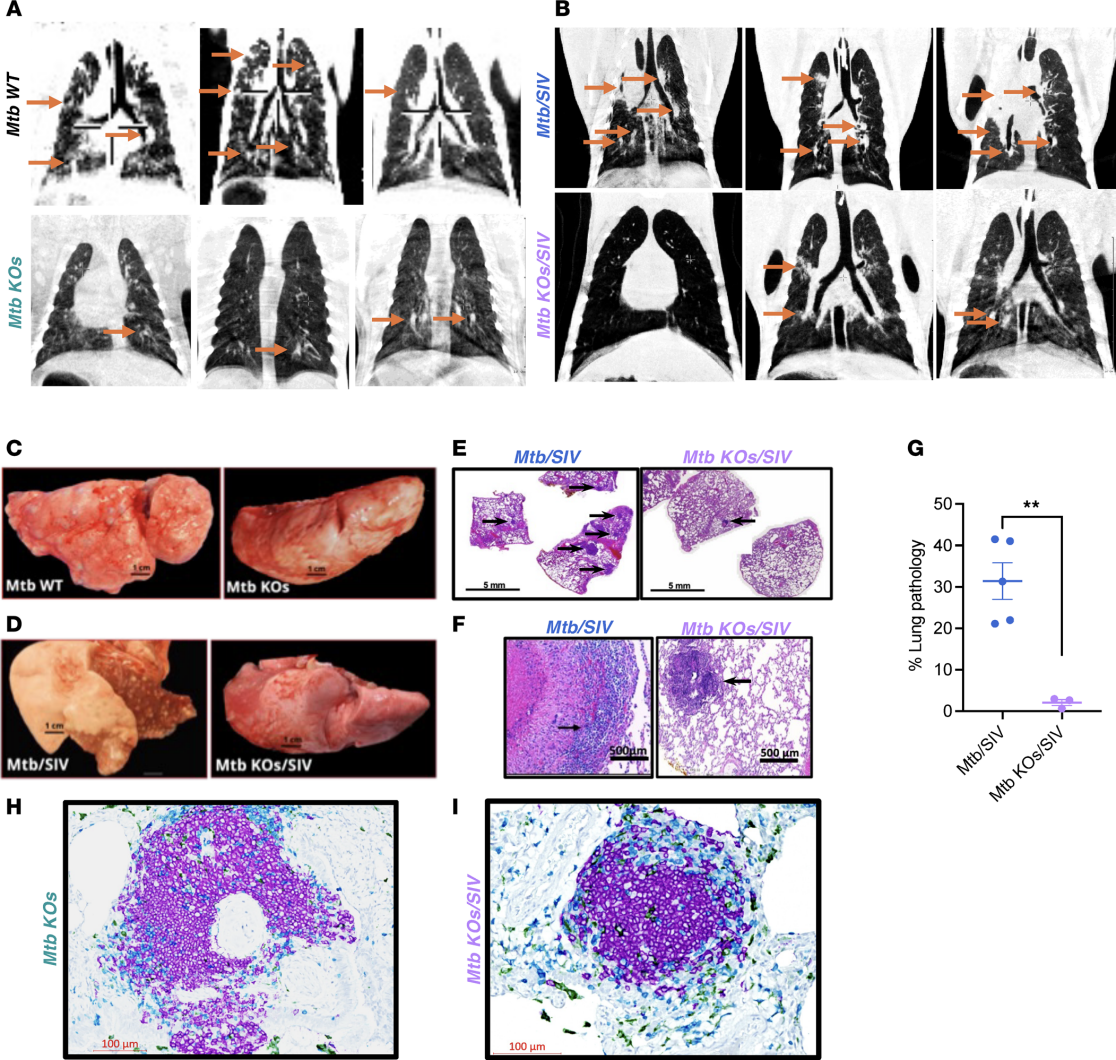
Lung pathology and TB lesions in *Mtb* KOs and *Mtb* KOs/SIV-cochallenged rhesus macaques. CT imaging of thoracic regions was performed on the macaques 4 weeks after *Mtb* KOs (**A**) and 4 weeks after SIV coinfection (**B**) prior to necropsies to examine the TB lesions. Representative images of lung tissues from macaques euthanized at 4 weeks after *Mtb* KOs (**C**) and 4 weeks after SIV coinfection (**D**), illustrating healthy gross pathology compared with the historical controls, are shown. To determine the effect of *Mtb* KOs/SIV coinfection on the lung pathology, lung tissue was collected at necropsy and subjected to H&E staining to study the cellular and granulomatous pathology. (**E**) A representative high-resolution photomicrograph is shown, and granulomatous lesions (2–4 mm) in these sections are marked by arrows. (**F**) H&E-stained lung sections from *Mtb* KOs/SIV-coinfected macaques show granuloma in *Mtb*/SIV-coinfected control animal and iBALT/iBALT-like structure in *Mtb* KOs/SIV-cochallenged animal, as indicated by arrows. (**G**) Percentage lung involvement was calculated by board-certified pathologist by quantification of the number of lesions per lobe of the lungs. Significance was determined using 1-way ANOVA with Tukey’s multiple comparisons test in GraphPad Prism v9.2.0. A *P* value < 0.05 was considered as statistically significant. **P < 0.01. Data are represented as mean ± SEM. Representative IHC staining lung sections from *Mtb* KOs-infected (**H**) and *Mtb* KOs/SIV-infected (**I**) macaques, highlighting staining with the iBALT markers — CD20 (purple), CD3 (teal), and CD68 (green). Images in panels **C**–**G** involving *Mtb*/SIV-coinfected and *Mtb* wild-type infected macaques are obtained from our previous publications (24, 81), as we did not perform experiments on this group in the current study.

It has been previously shown that *MtbΔsigH* induced minimal pathology, when aerosol-vaccinated in macaques despite SIV coinfection (24). Our detailed necropsies also showed that the *Mtb* KOs/SIV-challenged group exhibited substantially fewer pulmonary lesions and less TB-related pathology, as evident by the gross and histopathological analysis as well as computed tomography (CT) (Figure 5, A–E). CT was used to evaluate pulmonary pathology parameters in lungs, which were notably reduced in *Mtb* KOs and *Mtb* KOs/SIV group animals compared with those in *Mtb* wild-type and SIV-coinfected animals, respectively (Figure 5, A and B), suggesting that SIV-induced pathology was not exacerbated in *Mtb* KOs-challenged animals. As shown in Figure 5, C and D, we observed decreased gross pathology in lungs from *Mtb* KOs and *Mtb* KOs/SIV-infected animals in comparison with the control groups, *Mtb* wild-type, and *Mtb*/SIV-infected macaques, respectively. The lung tissues from the control group animals exhibited heavy tissue involvement with numerous tubercles (Figure 5, C and D). In contrast, lungs from *Mtb* KOs and *Mtb* KOs/SIV-infected animals exhibited reduced pathology with minimum involvement (Figure 5, C and D). In concordance, high-resolution scanning images showed a greater number of granulomas in sections from wild-type *Mtb*/SIV-coinfected animals in comparison with sections from *Mtb* KOs/SIV-coinfected macaques (Figure 5E and Supplemental Figure 5). In our histopathological analysis, we observed lesser tissue damage and reduced granuloma formation in sections from *Mtb* KOs and *Mtb* KOs/SIV-coinfected group (Figure 5F and Supplemental Figure 5). As shown in Figure 5F, severe granulomatous inflammation and loss of parenchymal space were observed in sections from *Mtb*/SIV-infected group. The detailed analysis of tissue damage in H&E-stained sections revealed that the extent of lung affected by TB lesions encompassed an average of 2% in the *Mtb* KOs/SIV group whereas it was ~31% in the case of *Mtb*/SIV-challenged animals (Figure 5G). Collectively, these observations suggest that *Mtb*Δ*sigH*-based KOs are safe and nonpathogenic in a stringent NHP model of TB.

### iBALT induction in MtbΔsigH-based KOs-challenged animals.

The observed granulomas in lungs from the *Mtb* KOs and SIV-coinfected macaques lacked the characteristic well-organized structure and appeared to be iBALTs. Thus, these lung sections were further analyzed and subjected to immunohistochemistry (IHC) staining for CD20^+^ B cells, CD3^+^ T cells, and CD68^+^ macrophage/dendritic cells followed by confocal imaging. As apparent in the analogous H&E-stained sections (Supplemental Figure 5), multiple organized lymphoid aggregates comprising B cells, T cells, and macrophages/monocytes were observed, consistent with the formation of iBALT structures (Figure 5, H and I). Moreover, the IHC staining revealed that the observed granulomas in the lungs of *Mtb* KOs and *Mtb* KOs/SIV-coinfected macaques were surrounded by well-organized iBALT structures per lesion (data not shown). As shown in Figure 5, H and I, these follicles were predominantly composed of CD20^+^ B cells, surrounded by spherical layers of CD3^+^ T cells, reinforcing previous findings that activated B cells are crucial for the control of TB in macaques (23, 64).

### MtbΔsigH-based KOs challenge induces antigen-specific T cell responses in airways.

**Figure 6:**
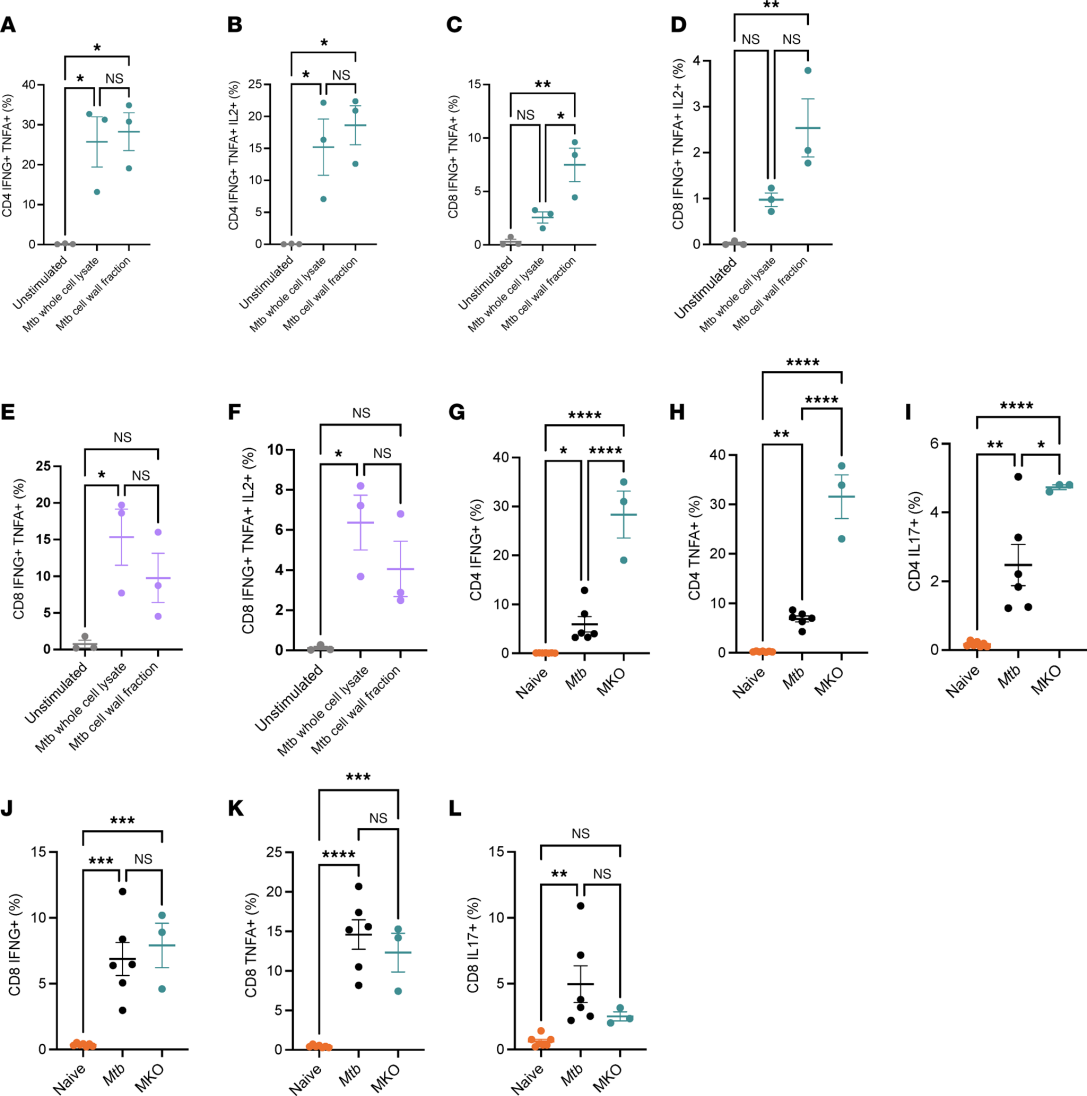
*MtbΔsigH-*based KOs induces superior antigen-specific immune responses in BAL. BAL cells collected at necropsy were stimulated with either whole cell or CW of *Mtb* or left unstimulated overnight for 16–18 hours. Activated cells were stained with different fluorophore-tagged antibodies (Supplemental Table 5), and the expression of surface markers was analyzed using flow cytometry. Frequencies of antigen-specific multifunctional CD4^+^ T cells expressing IFN-γ with TNF-α (**A**), IFN-γ, and TNF-α with IL-2 (**B**) and CD8^+^ T cells expressing IFN-γ with TNF-α (**C**), IFN-γ, and TNF-α with IL-2 (**D**) in BAL collected 4 weeks after *Mtb* KOs challenge is shown. Frequencies of multifunctional CD8^+^ T cells expressing IFN-γ with TNF-α (**E**) and IFN-γ, TNF-α with IL-2 (**F**) in BAL collected 8 weeks after *Mtb* KOs challenge (4 weeks after SIV challenge) is shown. Frequencies of antigen-specific multifunctional CD4^+^ T cells expressing IFN-γ (**G**), TNF-α (**H**), and IL-17 (**I**) and CD8^+^ T cells expressing IFN-γ (**J**), TNF-α (**K**), and IL-17 (**L**) in BAL collected 3–4 weeks postchallenge are shown for *Mtb* KOs (teal), *Mtb* (black), and naive animals (orange). Data involving *Mtb* wild-type infected macaques are obtained from our previous publications (77). Significance was determined using 1-way ANOVA with Tukey’s multiple comparisons test in GraphPad Prism v9.2.0. A *P* value < 0.05 was considered statistically significant. *P < 0.05; **P < 0.01; ***P < 0.001; ****P < 0.0001. The data shown are the mean ± SEM (*n* = 3).

Since *Mtb* is an intracellular pathogen with pulmonary pathology driven by IFN-γ, we next assessed whether the vaccination with *MtbΔsigH*-based KOs imparts protection against *M*. *tuberculosis*. Our previous results have conclusively shown *MtbΔsigH* to be immunogenic in rhesus and cynomolgus macaques, inducing robust *Mtb* antigen-specific T cell responses in airways, with peak responses observed at week 5 after intramucosal vaccination. Therefore, we also evaluated the antigen-specific T cell responses in BAL cells (Figure 6 and Supplemental Figures 6 and 7) and lungs (Supplemental Figures 6 and 8) isolated at week 4 after *MtbΔsigH*-based KOs and upon subsequent SIV challenge. As expected, intramucosal *Mtb* KOs challenge was found to be immunogenic in rhesus macaques, inducing antigen-specific multifunctional T cell responses in CD4^+^ (Figure 6, A and B) and CD8^+^ (Figure 6, C and D) compartments. Among the BAL CD4^+^ cells, almost 25%–30% were producing IFN-γ and TNF-α simultaneously in response to *Mtb* CDC1551 whole cell lysate (WCL) or *Mtb* CDC1551 cell wall fraction (CW), and 15% could produce IL-2 with IFN-γ and TNF-α simultaneously (Figure 6, A and B). In the CD8^+^ compartment, 2.5% of cells were responding to WCL and 7.5% to CW by producing IFN-γ and TNF-α simultaneously (Figure 6C). We found 1% of the BAL CD8^+^ T cells were producing IL-2 with IFN-γ and TNF-α simultaneously in response to WCL while a higher fraction of 2.5% were responsive to CW (Figure 6D). As expected, multifunctional responses to both WCL and CW were similar in the CD4^+^ compartment while CD8 multifunctionality was higher in CW due to presence of CD8^+^ dominant antigens like CFP10. In the CD4^+^ compartment, 25% stained positive for IFN-γ, 30% for TNF-α, 18%–20% for IL-2, and 4% for IL-17 in response to WCL and CW (Supplemental Figure 7, A–E). In the CD8^+^ compartment, 2.5% stained positive for IFN-γ, 5% for TNF-α, 1.5% for IL-2, and 1% for IL-17 in response to WCL while 7.5% stained positive for IFN-γ, 12% for TNF-α, 3% for IL-2, and 2.5% for IL-17 in response to CW (Supplemental Figure 7, F–J). At week 8 after *Mtb* KOs challenge/week 4 after SIV challenge, significant CD4^+^ T cell depletion was observed as expected (Figure 3G). However, the *Mtb* WCL-specific responses, including the multifunctional T cell responses, were retained in the CD8^+^ compartment (Figure 6, E and F, and Supplemental Figure 7, K–O). When compared with naive and wild-type *Mtb*-challenged controls, superior antigen-specific CD4^+^ (Figure 6, G–I) and CD8^+^ (Figure 6, J–L) T cell responses were induced by *Mtb* KOs challenge. In conclusion, challenge with pooled *MtbΔsigH-*based KOs elicited impressive protective multifunctional helper and cytotoxic T cell responses in the airways, further strengthening their feasibility as a safe and effective vaccine against TB.

## Discussion

While impressive gains have been made in the control of HIV globally, particularly after the advent of antiretroviral therapies, similar progress has been lacking with respect to TB (1). It is universally acknowledged that prevention via one or more effective, novel vaccines is the strategy likely to have the greatest impact on the TB pandemic (65). Despite WHO’s call for urgent action, progress in TB vaccine development has been less than desirable. Even presently, only a handful of new TB vaccine candidates are in advanced clinical trials (66). The concept of live attenuated vaccines that are based on *Mtb* was earlier theorized in the late 20th century, when advances in molecular biology techniques allowed rational design of attenuated *Mtb* strains by deleting genes encoding virulence factors. This led to the development of new live attenuated vaccine (LAV) candidates like MTBVAC (67). These genetically modified *Mtb* strains require at least 2 attenuating gene mutations to prevent reversal to the original virulent phenotype. Thus, MTBVAC, the only LAV strain that is currently in advanced TB clinical trials, carries deletions in the transcription factor *phoP* and the lipid biosynthesis gene *fadD26* (67, 68). An ideal TB vaccine should be nonpathogenic in animal models but provide significantly better immunogenicity and protection than BCG at the same time. LAV candidates are most likely among different classes of TB vaccine candidates to impart superior protection by virtue of expressing a complete repertoire of genes that encode immunodominant antigens. We have earlier demonstrated that *MtbΔsigH* protects 2 species of NHPs (rhesus and cynomolgus macaques) from exacerbated TB (15, 16, 23). In the current study, we developed 8 live rationally attenuated derivatives of *MtbΔsigH* CDC1551 as potent vaccine candidates against TB. All the mutant strains contain at least 2 independent stable gene mutations, in accordance with the guidelines established in the Second Geneva Consensus document for progressing new live mycobacterial vaccines to advanced clinical development. Our results suggest that at least 7 of these strains (except *ΔsigHΔmce1A*) fail to persist long-term in macaque lungs. Furthermore, these 7 strains are also safe in a model of *Mtb*/HIV coinfection.

A key lesson learned from the failure of subunit vaccine MVA85A is the importance of conducting detailed and uncompromising preclinical evaluations of new vaccine candidates in models that closely replicate the conditions intended for clinical testing (69–71). In this study, we have shown that the inactivation of various selected genes separately, in *MtbΔsigH* background, could serve as potentially safe TB vaccine candidates even in the setting of HIV coinfection using our macaque model of HIV coinfection. We have previously established that direct aerosol delivery of *MtbΔsigH* into macaque lungs elicited strong immune responses and provided remarkable protection against lethal TB (15, 16). Several studies have implicated the role of SigH in response to hostile stress conditions encountered by *Mtb* inside the host (17–22). Thus, the deletion of *sigH* has been strongly associated with the induction of protective immune responses (14–16, 23, 24). Aerosol vaccination with *Mtb*Δ*sigH* provides robust protection against lethal TB in both rhesus and cynomolgus macaques, demonstrating significantly greater efficacy than BCG vaccination (15, 16).

Using temperature-sensitive mycobacteriophages, we constructed *Mtb*-mutant strains harboring deletions in *secA2*, *hadC*, *mce1A*, *MT3785*, *metA*, *leuD*, *fbpA*, and *sapM* or *mce4E* and m*ce4F*. The growth patterns of the double-knockout (DKO) and triple-knockout (TKO) strains were comparable to the parental strain. Deletions of most of the selected genes did not alter the colony morphology or affect growth kinetics of *Mtb* in vitro. *MtbΔhadCΔsigH* exhibited a revival-defective phenotype and a lagged growth pattern compared with the parental strain, which may be attributed to the susceptibility of *MtbΔhadC* to cold shock, as previously reported (57). This strain also displayed an altered colony morphology. These findings align with previous observations that *hadC* mutant exhibits a unique mycolic acid profile and distinct colony morphology, suggesting alterations in cell surface properties. *MtbΔhadC* has also been shown to be less aggregated, more resistant to detergent, and impaired for biofilm development and sliding motility (57, 58).

The development of *Mtb* biofilms involves a series of steps starting from attachment of bacilli to the surface followed by sessile growth, matrix production, and dispersal (72, 73). There are various molecules including polysaccharides, structural proteins, mycolic acid, DNA, and GroEL1 chaperones and environmental factors that can regulate biofilm production (74, 75). Our observation that all the *Mtb* KOs including *MtbΔsigH* demonstrated a remarkable loss of biofilm formation in vitro points toward the crucial role SigH plays in controlling one or more of these factors. Importantly, *Mtb*’s ability to form biofilms in vivo, particularly at the margins of granuloma near B and T cells, enables them to evade immune defenses and resist antibiotic treatment (76). The observed biofilm defect in our *Mtb* KOs further strengthens the evidence that *MtbΔsigH*-based mutant strains are safe in vivo. TB drug susceptibility assays showed no difference among the knockout strains, indicating that their sensitivity to TB drugs remained unchanged by the genetic alterations.

To test the safety of these DKOs/TKOs in the setting of HIV coinfection, macaques were aerosol-vaccinated with 60–100 CFU of each strain. This dose of *MtbΔsigH* has been demonstrated to establish a nonpathogenic infection in macaques and provide exceptional protection against a subsequent lethal challenge when used as a vaccine (15, 24). To contextualize our findings, we compared the results with control data obtained from our prior studies using the similar experimental setup. Animals from *Mtb* wild-type group were administered 100–200 CFU *Mtb* CDC1551 via aerosol as this dose typically induces TB in 100% of the exposed rhesus macaques (77). To establish latent TB, macaques in the *Mtb*/SIV group were infected with 10 CFU of *Mtb* CDC1551, followed by intravenous administration of 300 TCID_50_ SIV, to induce reactivation of the infection (78). Our results demonstrate that all the macaques vaccinated with a mix of DKOs/TKOs remain healthy with no mortality or disease-associated symptoms till the endpoint of the study, despite presence of pathogenic replicating SIV in the bloodstream. CRP data of all the animals are suggestive of the low likelihood of acute inflammatory conditions, such as bacterial infections or tissue injury. In contrast, macaques coinfected with wild-type *Mtb* and SIV that reactivate from latent TB exhibit severe pathology associated with both SIV and TB (78). Moreover, these reactivated macaques exhibit high bacterial loads in the lungs, increased extrapulmonary dissemination, and severe TB granuloma pathology. In contrast, fewer bacilli could be cultured in the lungs and bronchial lymph nodes, while culturable bacilli were completely absent in the extrapulmonary tissues, in the *Mtb* KOs/SIV-infected macaque group. These findings are consistent with previously reported data on *MtbΔsigH* (24). In agreement with the CT and pathology scores, we observed substantially reduced tissue damage in lung sections from macaques infected with the *Mtb* KOs in comparison with the wild-type strain–infected animals as evident from the histological examination data.

A characteristic feature of protection imparted by *MtbΔsigH* is the significant recruitment of profound iBALT (15, 16, 24). iBALTs resemble secondary lymphoid organs in structure and function and play a role in local immune responses by supporting antigen presentation, lymphocyte activation, and adaptive immunity within pulmonary tissue. Consistent with the previous findings with *MtbΔsigH*, we observed that lungs from *Mtb* KOs-vaccinated and *Mtb* KOs/SIV-coinfected macaques contained several iBALT lesions particularly in the vicinity of granulomas. Furthermore, the presence of CD20^+^ B cell–dominant aggregates in the center of these follicles surrounded by an outer layer of T cells suggests site-specific immune activation induced by *MtbΔsigH*-based KOs. This interplay between B/T cells is pivotal for the protection against TB. Further analysis using techniques such as cyclic immunofluorescence-based multiplex spatial biology staining of lung sections would help distinguish specific immune cell subsets and provide deeper insight into these findings. Overall, these results advocate for the safety of *MtbΔsigH*-based DKO/TKO not only in immunocompetent but also in immunocompromised SIV-coinfected macaques.

*MtbΔsigH*-based KOs induced strong protective T cell responses in lungs. Our work has previously demonstrated that aerosol vaccination with *MtbΔsigH* elicits strong local innate and adaptive immune responses resulting in significantly higher recruitment of lung-homing, Th1/Th17 or Tc1/Th17 T cells as well as B cells to the site of infection (15, 16, 23). These lymphocytes are type I IFN conditioned and express significantly higher levels of protective cytokines in response to *Mtb* rechallenge (16). The expression of these key cytokines primes infected as well as bystander macrophages to control *Mtb* infection at a significantly better level. Importantly, *MtbΔsigH*-based KOs retain these features of protective immune responses, including induction of iBALTs and polyfunctional T cell responses.

Our observation on active replication of *MtbΔsigHΔmce1A* strain in the lungs and upper respiratory tract (BAL) is consistent with the previous studies that have confirmed Δ*mce1A* strain of *Mtb* overgrows the wild-type *Mtb* in BALB/c mice, demonstrating the hypervirulence phenotype of the mutant strain (63). Furthermore, mouse peritoneal macrophages infected with the *Δmce1* mutant produced less TNF-α, IL-6, MCP-1, and NO but not IL-4 than with wild-type (63). Our current observation that *MtbΔsigHΔmce1* (but not the other 7 strains) was able to replicate in immunocompetent macaque lungs further highlights the robust protective effect of the lack of a functional SigH regulon. Despite active replication of the *MtbΔsigHΔmce1* mutant in the lungs and thoracic regions of *Mtb* KOs-vaccinated animals, these macaques remained healthy and free from TB and TB/SIV-associated pathology. These results also likely indicate that a live-attenuated MTBVAC approach containing *Δmce1* may be infeasible.

The current study had some limitations, in particular, the short duration of monitoring the animals after SIV, but these early-stage preclinical findings demonstrate the favorable immunogenicity and safety profiles of *MtbΔsigH-*based vaccine candidates in rhesus macaques. Additionally, our approach to assess the growth phenotype of the 8 mutant strains containing *ΔsigH* deletion in combination resulted in the use of fewer rhesus macaques, and we were able to identify bacilli cultured in both immunocompetent as well as SIV-coinfected macaque groups as primarily (91%) *ΔsigHΔmce1*. A limitation of this approach, however, is that immunogenicity data from each individual strain are difficult to glean. Thus, future experiments should evaluate the efficacy and protection imparted by each of the 7 DKO/TKO strains (excluding *MtbΔsigHΔmce1A*) against active lethal *Mtb* challenge, as well as preventing reactivation from latent tuberculosis infection using the rhesus macaque *Mtb*/SIV coinfection model (78–80) relative to BCG vaccination. In conclusion, our findings demonstrate the safety of *MtbΔsigH*-derived DKOs and TKOs and strongly support further clinical development with an aim to facilitate the advancement of these candidates to clinical trials.

## Methods

### Sex as a biological variable.

Our study examined male and female animals, and similar findings are reported for both sexes.

### Bacterial strains, plasmids, and culture conditions.

*Mtb* strain CDC1551 (NR-13649) and *M*. *smegmatis* mc2155 (ARP-2195) were obtained from BEI Resources. Primers used in the study are listed in Supplemental Table 2. The recombinant constructs and *Mtb* gene knockout strains were verified by DNA sequencing. Mycobacterial strains were cultured in Middlebrook (MB) 7H9 or 7H11 medium as per standard protocols. *MtbΔsigHΔmetA* and *MtbΔsigHΔleuD* strains were grown in media supplemented with l-methionine at 50 μg/mL and l-leucine at 50 μg/mL, respectively. When required, hygromycin was added at 75 μg/mL for *Mtb* or 150 μg/mL for *E*. *coli*. Kanamycin and apramycin were used at 25 μg/mL and 75 μg/mL, respectively, as needed. For biofilm formation, log phase cultures of various strains were diluted and grown in Sauton’s medium (72) in 6-well plates tightly sealed with PARAFILM (Parafilm M, Bemis Company, Inc.), at 37°C for 4-5 weeks.

### DNA manipulations.

Molecular biology was carried out as per manufacturer’s recommendations: oligo synthesis (Integrated DNA Technologies), DNA purification (QIAGEN and Promega), enzyme restrictions and T4 DNA ligase (New England Biolabs), PCR with the Platinum High Fidelity Taq DNA polymerase (Invitrogen), and pGEM-T Easy Vector Systems (Promega). DNA insertions were confirmed by sequencing (GENEWIZ).

### Construction of various knockout strains of M.

*tuberculosis*. *Mtb* KO mutants were generated in the parental *Mtb* CDC1551 strain using in vitro–generated, temperature-sensitive mycobacteriophages (55). Briefly, to delete individual genes, *sapM*, *hadC*, *MT3785*, *metA*, and *leuD*, as well as the *mce4E-mce4F* cluster, 700-800 bp upstream and downstream regions of each target gene were PCR amplified and cloned on either side of *hyg^R^* cassette into a cosmid vector, pYUB854, to generate allelic exchange sequences. The recombinant pYUB854-Δ*KOs* was *Pac*I digested and packaged into phage DNA, phAE159. The recombinant phagemids were electroporated in *M*. *smegmatis* to generate temperature-sensitive mycobacteriophages. These temperature-sensitive mycobacteriophages were used to transduce early-log phase cultures of *Mtb* CDC1551 (OD_600nm_ 0.3–0.4) to generate deletion strains. For the generation of *ΔsapMΔfbpA* DKO strain, the hygromycin resistance gene in pYUB854-Δ*fbpA::hyg^R^* was replaced with the kanamycin resistance gene, resulting in pYUB854-Δ*fbpA::kan^R^*. The recombinant cosmid, pYUB854-Δ*fbpA::kan^R^*, was packaged, and temperature-sensitive mycobacteriophages were prepared as described above. Early-log-phase cultures of *Mtb*Δ*sapM::hyg^R^* strain were transduced with temperature-sensitive Δ*fbpA::kan^R^* mycobacteriophages. *sigH* deletion in each of these mutant strains was accomplished using the same methodology with a slight modification: The hygromycin resistance cassette was replaced with an apramycin resistance gene. The transductants were selected on MB7H11 plates containing hygromycin, kanamycin, or apramycin. *ΔsecA2* and *Δmce1A* recombinant phagemids, provided by William Jacobs (Albert Einstein College of Medicine, New York, New York, USA), were used to introduce these gene deletions (56). These deletions were unmarked using a second transduction step using another phage, phAE280 (56). The unmarked deletions and replacement of various genes by the *hyg^R^*/*kan^R^*/*apra^R^*, in individual KOs, were verified by performing PCR on lysates of recovered clones as well as by sequencing.

### NHP study design and infections.

All procedures adhered to NIH guidelines and received approval from the Institutional Animal Care and Use Committees of Texas Biomedical Research Institute. A total of 6 specific pathogen-free tuberculin skin test–negative (TST^–^) Indian-origin rhesus macaques (*Macaca mulatta*) obtained from California National Primate Research Center (CNPRC), were used in this study protocol (Supplemental Table 3). All 6 macaques were aerosol vaccinated in a manner designed to deposit 50-100 CFUs of each of the *Mtb*Δ*sigH*-based knockout strains, as described earlier (15). At 4 weeks postvaccination, 3 out of 6 macaques were coinfected with 300 TCID_50_ SIVmac_239_ (provided by the Preston Marx/Nick Manness Laboratory, Tulane National Primate Research Center, Covington, Louisiana, USA) via the intravenous route as described earlier (80–82). TST was performed at weeks 3 and 5 after TB infection to confirm infection. All the macaques were monitored for CRP, percentage body weight, and body temperature weekly as well as BAL CFUs and CT scans throughout the study period (Figure 3A). Dissemination was evaluated during necropsy by culturing lungs; bronchial, axillary, and mesenteric lymph nodes; as well as spleen, liver, and kidney tissues to measure CFUs. Demographic information including age, sex, etc. and study-specific information of macaques are provided (Supplemental Table 3). A total of 3 animals were euthanized at 4 weeks postvaccination and remaining 3 were euthanized at week 4 after SIV challenge. The *Mtb* wild-type infected as well as *MtbΔsigH*/SIV and *Mtb*/SIV coinfection groups utilized data/images from our previous publication (24, 77, 78, 81, 83), in order to reduce the use of NHPs in research.

### Sampling.

TST was performed 1–3 weeks before challenge and at weeks 3 and 5 postchallenge as well as at endpoint, as described (15). CT scans were performed at 4 weeks after *Mtb* KOs vaccination and 4 weeks after SIV infection as described. BAL samples were obtained 1 week before *Mtb* KOs vaccination or SIV infection and subsequently every 2 weeks, as described (15, 16). BAL cells were used for determining bacterial burden as described (15, 16). Blood samples were collected 1 week prior to vaccination or SIV infection and thereafter on a weekly basis, for measuring complete blood count, serum chemistry, including serum CRP, as described (15, 16).

### Tissue bacterial burden and pathology.

Tissues were collected and processed as described (15, 16). CFUs were determined per gram of tissue and per milliliter of BAL fluid. Lung pathology at necropsy was assessed by a board-certified veterinary pathologist in a blinded manner, utilizing zinc-formalin-fixed paraffin-embedded (FFPE) tissues representing all lung lobes using previously described methods (15, 16).

### Computed tomography imaging.

Lung field CT images were acquired using Multiscan LFER150 PET/CT (MEDISO) scanner as previously described (83–86). 3D ROI Tools available in Vivoquant (Invicro) were used for image analysis (85). The ventral lung lobes were described as caudal, and the upper lung lobes were described as cranial. The CT resolution was fair with moderate beam hardening/streak artifacts due to cone beam technology. Axial/transverse reconstruction series were provided in soft tissue windows. The studies were reviewed using Sectra IDS7 viewing software in a lung window with centerline –230.0 and window of 2,250.0.

### Viral load measurement.

Viral loads in acellular BAL supernatant and plasma were determined by quantitative reverse transcription PCR at necropsy (4 weeks after SIV) (82–84). A lower limit of 100 copies/sample was set for quantification of SIV copies in this assay.

### Immune response analysis.

Different immunocyte populations were quantified and characterized in BAL and lungs using flow cytometry, following established protocols (16, 86). T cell populations and their functionality were assessed through stimulations and analyzed using flow cytometry (Supplemental Tables 4 and 5), as detailed in prior publications (16).

### Immunohistochemistry staining.

Fluorescent immunohistochemistry was performed on FFPE lung tissues. Briefly, 4 μm–thick FFPE tissue sections were cut, mounted onto positively charged slides, and allowed to air-dry overnight. Slides were then loaded onto the Roche Ventana Discovery ULTRA IHC/ISH automated stainer for the detection of the CD3, CD20, and CD68 protein markers. Deparaffinization was performed using Discovery Wash (Roche), and cell conditioning was performed using Discovery CC1 (Roche) at 95°C for 64 minutes. The endogenous peroxidase was blocked using Discovery Inhibitor (Roche) for 8 minutes. Slides were then incubated with anti-CD68 (KP-1) prediluted antibody for 20 minutes at 36°C, and its detection was performed by using Discovery OmniMap anti-Ms HRP (Roche) for 16 minutes at 36°C. CD68 was visualized by applying Discovery Green HRP chromogen kit (Roche) for 32 minutes. Slides were then denatured using ULTRA Cell Conditioning Solution (ULTRA CC2, Roche) at 95°C for 8 minutes. Next, slides were incubated with anti-CD20 (L26) prediluted antibody for 16 minutes at 36°C and was detected using Discovery OmniMap anti-Ms HRP for 16 minutes at 36°C. Visualization of CD20 (L26) was achieved by applying Discovery Purple chromogen kit (Roche) for 32 minutes. Slides were then denatured once more using ULTRA CC2 at 95°C for 8 minutes, then incubated with anti-CD3 (2GV6) prediluted antibody for 20 minutes at 36°C. Detection was performed using Anti-rabbit HQ (Roche) for 8 minutes at 36°C followed by Anti-HQ HRP (Roche) for 8 minutes at 36°C. Visualization was achieved by applying Discovery Teal HRP chromogen kit (Roche) to the slides for 32 minutes. The slides were then counterstained using hematoxylin for 12 minutes followed by Bluing Reagent (Roche) for 8 minutes. The stained slides were scanned in the Axio Scan Z1 (Zeiss), and the images were analyzed using HALO software as previously described (16, 87).

### Statistics.

Statistical analysis was performed using an unpaired 2-tailed Student’s *t* test and 1- or 2-way ANOVA with Holm-Šídák or Tukey’s correction as applicable in GraphPad Prism (version 9.2.0). A *P* value < 0.05 was considered statistically significant. Data are represented as mean ± SEM.

### Study approval.

All infected macaques were housed under Animal Biosafety Level 3 facilities at the Southwest National Primate Research Center, where they were treated according to the standards recommended by Association for Assessment and Accreditation of Laboratory Animals International and the NIH *Guide for the Care and Use of Laboratory Animals* (National Academies Press, 2011). Ethics approval for the study procedures was obtained from the Institutional Animal Care and Use Committee and Recombinant DNA Committee at the Texas Biomedical Research Institute.

### Data availability.

Supporting Data Values are provided with this manuscript in XLS file format. All data supporting the findings of this study are available within this manuscript and its supplement.

## Author contributions

DK conceived the idea and supervised the study. DK, GA, DKS, SM, MA, CJ, and SAK designed the study. GA constructed the knockout strains with assistance from DKS and was responsible for conducting, coordinating, and managing the safety study in macaques. GA analyzed the clinical and bacterial data. GA and DKS analyzed the flow cytometry data. CWM assisted with PCR-based screening. DKS, AD, VSRD, and BA assisted with necropsy sampling. XA performed CT imaging. DK and DKS contributed to data interpretation. GA wrote the manuscript with inputs from DK, DKS, SAK, and CJ. ED, ODG, and VS performed the necropsies and histopathology analysis. VS performed the scanning of the confocal slides. SHU was the attending veterinarian on the study.

## Supplementary Material

Supplemental data

Unedited blot and gel images

Supplemental tables 1-5

Supporting data values

## Figures and Tables

**Table 1:**
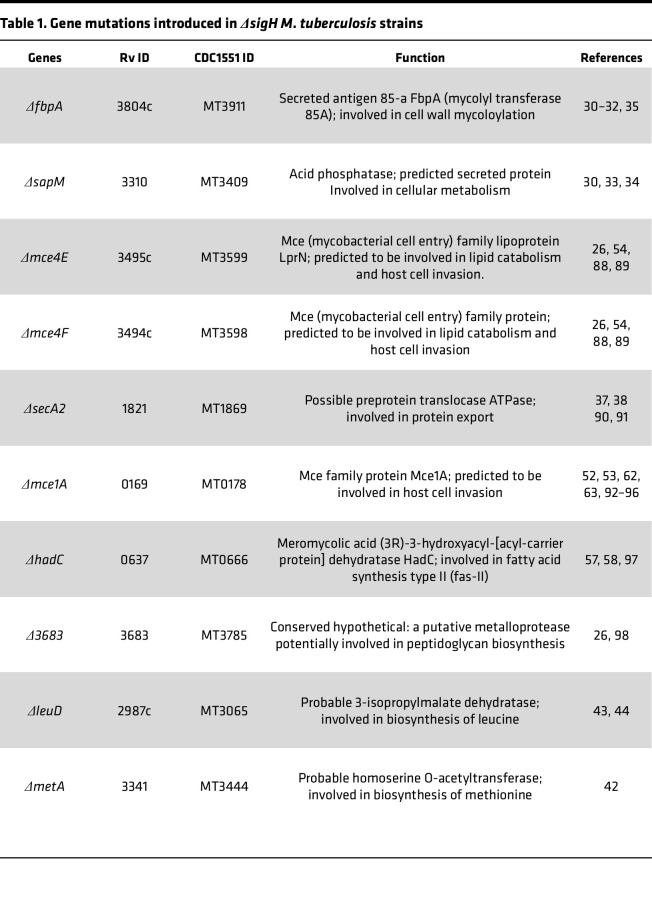
Gene mutations introduced in *ΔsigH* *M*. *tuberculosis* strains

## Version 1 08/28/2025

In-Press Preview

## Version 2 10/08/2025

Electronic publication

## References

[1] Bagcchi S (2023). WHO’s global tuberculosis report 2022. Lancet Microbe.

[2] https://www.who.int/publications/i/item/WHO-HTM-TB-2015.19.

[3] McShane H (2019). Insights and challenges in tuberculosis vaccine development. Lancet Respir Med.

[4] Kaufmann SH (2010). Future vaccination strategies against tuberculosis: thinking outside the box. Immunity.

[5] Ottenhoff TH, Kaufmann SH (2012). Vaccines against tuberculosis: where are we and where do we need to go?. PLoS Pathog.

[6] Portnoy A (2023). The cost and cost-effectiveness of novel tuberculosis vaccines in low- and middle-income countries: a modeling study. PLoS Med.

[7] Fine PE (1995). Variation in protection by BCG: implications of and for heterologous immunity. Lancet.

[8] Gengenbacher M (2017). BCG - old workhorse, new skills. Curr Opin Immunol.

[9] Sambandamurthy VK (2005). Live attenuated mutants of Mycobacterium tuberculosis as candidate vaccines against tuberculosis. Microbes Infect.

[10] Martinot AJ (2020). Protective efficacy of an attenuated Mtb ΔLprG vaccine in mice. PLoS Pathog.

[11] Gonzalo-Asensio J (2017). MTBVAC: attenuating the human pathogen of tuberculosis (TB) toward a promising vaccine against the TB epidemic. Front Immunol.

[12] Brennan MJ (2004). Tuberculosis vaccine development: research, regulatory and clinical strategies. Expert Opin Biol Ther.

[13] Martin C (2021). MTBVAC, a live TB vaccine poised to initiate efficacy trials 100 years after BCG. Vaccine.

[14] Mehra S (2012). The Mycobacterium tuberculosis stress response factor SigH is required for bacterial burden as well as immunopathology in primate lungs. J Infect Dis.

[15] Kaushal D (2015). Mucosal vaccination with attenuated Mycobacterium tuberculosis induces strong central memory responses and protects against tuberculosis. Nat Commun.

[16] Singh DK (2025). Prevention of tuberculosis in cynomolgus macaques by an attenuated Mycobacterium tuberculosis vaccine candidate. Nat Commun.

[17] Mehra S, Kaushal D (2009). Functional genomics reveals extended roles of the Mycobacterium tuberculosis stress response factor sigmaH. J Bacteriol.

[18] Raman S (2001). The alternative sigma factor SigH regulates major components of oxidative and heat stress responses in Mycobacterium tuberculosis. J Bacteriol.

[19] Rohde KH (2007). Mycobacterium tuberculosis invasion of macrophages: linking bacterial gene expression to environmental cues. Cell Host Microbe.

[20] Rustad TR (2008). The enduring hypoxic response of Mycobacterium tuberculosis. PLoS One.

[21] Graham JE, Clark-Curtiss JE (1999). Identification of Mycobacterium tuberculosis RNAs synthesized in response to phagocytosis by human macrophages by selective capture of transcribed sequences (SCOTS). Proc Natl Acad Sci U S A.

[22] Schnappinger D (2003). Transcriptional adaptation of Mycobacterium tuberculosis within macrophages: insights into the phagosomal environment. J Exp Med.

[23] Swanson RV (2023). Antigen-specific B cells direct T follicular-like helper cells into lymphoid follicles to mediate Mycobacterium tuberculosis control. Nat Immunol.

[24] Foreman TW (2017). Nonpathologic infection of macaques by an attenuated mycobacterial vaccine is not reactivated in the setting of HIV co-infection. Am J Pathol.

[25] Kamath AT (2005). New live mycobacterial vaccines: the Geneva consensus on essential steps towards clinical development. Vaccine.

[26] Dutta NK (2010). Genetic requirements for the survival of tubercle bacilli in primates. J Infect Dis.

[27] Chen ZW (1997). Disseminated granulomatous disease in a simian immunodeficiency virus- and bacille Calmette-Guèrin-infected rhesus monkey. AIDS.

[28] Dutta NK (2012). The stress-response factor SigH modulates the interaction between Mycobacterium tuberculosis and host phagocytes. PLoS One.

[29] Walker KB (2010). The second Geneva consensus: recommendations for novel live TB vaccines. Vaccine.

[30] Saikolappan S (2012). The fbpA/sapM double knock out strain of Mycobacterium tuberculosis is highly attenuated and immunogenic in macrophages. PLoS One.

[31] Copenhaver RH (2004). A mutant of Mycobacterium tuberculosis H37Rv that lacks expression of antigen 85A is attenuated in mice but retains vaccinogenic potential. Infect Immun.

[32] Armitige LY (2000). Disruption of the genes encoding antigen 85A and antigen 85B of Mycobacterium tuberculosis H37Rv: effect on growth in culture and in macrophages. Infect Immun.

[33] Vergne I (2005). Mechanism of phagolysosome biogenesis block by viable Mycobacterium tuberculosis. Proc Natl Acad Sci U S A.

[34] Saleh MT, Belisle JT (2000). Secretion of an acid phosphatase (SapM) by Mycobacterium tuberculosis that is similar to eukaryotic acid phosphatases. J Bacteriol.

[35] Katti MK (2008). The Delta fbpA mutant derived from Mycobacterium tuberculosis H37Rv has an enhanced susceptibility to intracellular antimicrobial oxidative mechanisms, undergoes limited phagosome maturation and activates macrophages and dendritic cells. Cell Microbiol.

[36] Edwards KM (2001). Iron-cofactored superoxide dismutase inhibits host responses to Mycobacterium tuberculosis. Am J Respir Crit Care Med.

[37] Sullivan JT (2012). The Mycobacterium tuberculosis SecA2 system subverts phagosome maturation to promote growth in macrophages. Infect Immun.

[38] Augenstreich J, Briken V (2020). Host cell targets of released lipid and secreted protein effectors of Mycobacterium tuberculosis. Front Cell Infect Microbiol.

[39] Sadagopal S (2009). Reducing the activity and secretion of microbial antioxidants enhances the immunogenicity of BCG. PLoS One.

[40] Jensen K (2013). A neonatal oral Mycobacterium tuberculosis-SIV prime / intramuscular MVA-SIV boost combination vaccine induces both SIV and *Mtb*-specific immune responses in infant macaques. Trials Vaccinol.

[41] Hondalus MK (2000). Attenuation of and protection induced by a leucine auxotroph of Mycobacterium tuberculosis. Infect Immun.

[42] Berney M (2015). Essential roles of methionine and S-adenosylmethionine in the autarkic lifestyle of Mycobacterium tuberculosis. Proc Natl Acad Sci U S A.

[43] Sampson SL (2011). Extended safety and efficacy studies of a live attenuated double leucine and pantothenate auxotroph of Mycobacterium tuberculosis as a vaccine candidate. Vaccine.

[44] Sampson SL (2004). Protection elicited by a double leucine and pantothenate auxotroph of Mycobacterium tuberculosis in guinea pigs. Infect Immun.

[45] Larsen MH (2009). Efficacy and safety of live attenuated persistent and rapidly cleared Mycobacterium tuberculosis vaccine candidates in non-human primates. Vaccine.

[46] Sambandamurthy VK (2005). Long-term protection against tuberculosis following vaccination with a severely attenuated double lysine and pantothenate auxotroph of Mycobacterium tuberculosis. Infect Immun.

[47] Sassetti CM, Rubin EJ (2003). Genetic requirements for mycobacterial survival during infection. Proc Natl Acad Sci U S A.

[48] Lamichhane G (2005). Designer arrays for defined mutant analysis to detect genes essential for survival of Mycobacterium tuberculosis in mouse lungs. Infect Immun.

[49] Rengarajan R (2005). Effect of disorder on the optical properties of colloidal crystals. Phys Rev E Stat Nonlin Soft Matter Phys.

[50] Griffin JE (2011). High-resolution phenotypic profiling defines genes essential for mycobacterial growth and cholesterol catabolism. PLoS Pathog.

[51] Griffin JE (2012). Cholesterol catabolism by Mycobacterium tuberculosis requires transcriptional and metabolic adaptations. Chem Biol.

[52] Forrellad MA (2014). Role of the Mce1 transporter in the lipid homeostasis of Mycobacterium tuberculosis. Tuberculosis (Edinb).

[53] Gioffre A (2005). Mutation in mce operons attenuates Mycobacterium tuberculosis virulence. Microbes Infect.

[54] Pandey AK, Sassetti CM (2008). Mycobacterial persistence requires the utilization of host cholesterol. Proc Natl Acad Sci U S A.

[55] Bardarov S (2002). Specialized transduction: an efficient method for generating marked and unmarked targeted gene disruptions in Mycobacterium tuberculosis, M. bovis BCG and M. smegmatis. Microbiology (Reading).

[56] Jain P (2014). Specialized transduction designed for precise high-throughput unmarked deletions in Mycobacterium tuberculosis. mBio.

[57] Jamet S (2015). The non-essential mycolic acid biosynthesis genes hadA and hadC contribute to the physiology and fitness of Mycobacterium smegmatis. PLoS One.

[58] Slama N (2016). The changes in mycolic acid structures caused by hadC mutation have a dramatic effect on the virulence of Mycobacterium tuberculosis. Mol Microbiol.

[59] Ojha AK (2008). Growth of Mycobacterium tuberculosis biofilms containing free mycolic acids and harbouring drug-tolerant bacteria. Mol Microbiol.

[60] Richards JP (2019). Adaptation of Mycobacterium tuberculosis to biofilm growth is genetically linked to drug tolerance. Antimicrob Agents Chemother.

[61] Assefa M, Girmay G (2024). Mycobacterium tuberculosis biofilms: immune responses, role in TB pathology, and potential treatment. Immunotargets Ther.

[62] Nazarova EV (2017). Rv3723/LucA coordinates fatty acid and cholesterol uptake in Mycobacterium tuberculosis. Elife.

[63] Shimono N (2003). Hypervirulent mutant of Mycobacterium tuberculosis resulting from disruption of the mce1 operon. Proc Natl Acad Sci U S A.

[64] Phuah JY (2012). Activated B cells in the granulomas of nonhuman primates infected with Mycobacterium tuberculosis. Am J Pathol.

[65] Knight GM (2014). Impact and cost-effectiveness of new tuberculosis vaccines in low- and middle-income countries. Proc Natl Acad Sci U S A.

[66] Da Costa C (2024). Perspectives on development and advancement of new tuberculosis vaccines. Int J Infect Dis.

[67] Arbues A (2013). Construction, characterization and preclinical evaluation of MTBVAC, the first live-attenuated M. tuberculosis-based vaccine to enter clinical trials. Vaccine.

[68] Aguilo N (2016). MTBVAC vaccine is safe, immunogenic and confers protective efficacy against Mycobacterium tuberculosis in newborn mice. Tuberculosis (edinb).

[69] Tameris MD (2013). Safety and efficacy of MVA85A, a new tuberculosis vaccine, in infants previously vaccinated with BCG: a randomised, placebo-controlled phase 2b trial. Lancet.

[70] Beverley P (2013). Environmental effects on protection against Mycobacterium tuberculosis after immunization with Ad85A. Vaccine.

[71] Kashangura R (2015). Effects of MVA85A vaccine on tuberculosis challenge in animals: systematic review. Int J Epidemiol.

[72] Kulka K (2012). Growth of Mycobacterium tuberculosis biofilms. J Vis Exp.

[73] Richards JP, Ojha AK (2014). Mycobacterial biofilms. Microbiol Spectr.

[74] Esteban J, Garcia-Coca M (2017). Mycobacterium biofilms. Front Microbiol.

[76] Chakraborty P (2021). Biofilm formation in the lung contributes to virulence and drug tolerance of Mycobacterium tuberculosis. Nat Commun.

[77] Sharan R (2021). Characterizing early T cell responses in nonhuman primate model of tuberculosis. Front Immunol.

[78] Foreman TW (2016). CD4+ T-cell-independent mechanisms suppress reactivation of latent tuberculosis in a macaque model of HIV coinfection. Proc Natl Acad Sci U S A.

[79] Mehra S (2011). Reactivation of latent tuberculosis in rhesus macaques by coinfection with simian immunodeficiency virus. J Med Primatol.

[80] Bucsan AN (2019). Mechanisms of reactivation of latent tuberculosis infection due to SIV coinfection. J Clin Invest.

[81] Sharan R (2022). Antiretroviral therapy timing impacts latent tuberculosis infection reactivation in a Mycobacterium tuberculosis/SIV coinfection model. J Clin Invest.

[82] Sharan R (2022). Antiretroviral therapy timing impacts latent tuberculosis infection reactivation in a tuberculosis/simian immunodeficiency virus coinfection model. J Clin Invest.

[83] Singh B (2024). Indoleamine-2,3-dioxygenase inhibition improves immunity and is safe for concurrent use with cART during Mtb/SIV coinfection. JCI Insight.

[84] Sharan R (2022). Isoniazid and rifapentine treatment effectively reduces persistent M. tuberculosis infection in macaque lungs. J Clin Invest.

[85] Singh B (2023). Inhibition of indoleamine dioxygenase leads to better control of tuberculosis adjunctive to chemotherapy. JCI Insight.

[86] Singh DK (2021). Responses to acute infection with SARS-CoV-2 in the lungs of rhesus macaques, baboons and marmosets. Nat Microbiol.

[87] Singh DK (2022). Myeloid cell interferon responses correlate with clearance of SARS-CoV-2. Nat Commun.

[88] Saini NK (2008). Characterization of Mce4A protein of Mycobacterium tuberculosis: role in invasion and survival. BMC Microbiol.

[89] Kumar A (2003). Analysis of expression profile of mammalian cell entry (mce) operons of Mycobacterium tuberculosis. Infect Immun.

[90] Converse SE, Cox JS (2005). A protein secretion pathway critical for Mycobacterium tuberculosis virulence is conserved and functional in Mycobacterium smegmatis. J Bacteriol.

[91] Jensen K (2012). A recombinant attenuated Mycobacterium tuberculosis vaccine strain is safe in immunosuppressed simian immunodeficiency virus-infected infant macaques. Clin Vaccine Immunol.

[92] Arruda S (1993). Cloning of an M. tuberculosis DNA fragment associated with entry and survival inside cells. Science.

[93] Chitale S (2001). Recombinant Mycobacterium tuberculosis protein associated with mammalian cell entry. Cell Microbiol.

[94] Klepp LI (2022). Mycobacterial MCE proteins as transporters that control lipid homeostasis of the cell wall. Tuberculosis (edinb).

[95] Casali N, Riley LW (2007). A phylogenomic analysis of the Actinomycetales mce operons. BMC Genomics.

[96] Chen J (2023). Structure of an endogenous mycobacterial MCE lipid transporter. Nature.

[97] Sacco E (2007). Rv3389C from Mycobacterium tuberculosis, a member of the (R)-specific hydratase/dehydratase family. Biochim Biophys Acta.

[98] Kaur P (2021). A multi-targeting pre-clinical candidate against drug-resistant tuberculosis. Tuberculosis (edinb).

