# Supplementary material for: Development and preclinical evaluation of next-generation ΔsigH-based live candidate vaccines
Source: JCI Insight. 2025 Aug 28;10(19):e195947. doi: 10.1172/jci.insight.195947 (PMC12513488; doi:10.1172/jci.insight.195947)
Supplement: Supplemental data [file jciinsight-10-195947-s237.pdf]

Supplementary information

Development and Preclinical Evaluation of Next-generation *DsigH*-based Live Candidate Vaccines

Authors: Garima Arora[1], Caden W. Munson[1], Mushtaq Ahmed[2], Vinay Shivanna[1], Annu Devi[1], Venkata S. R. Devireddy[1], Basil Antony[1], Shannan Hall-Ursone[1], Olga D. Gonzalez[1], Edward J. Dick Jr[1], Chinnaswamy Jagannath[3], Xavier Alvarez[1], Smriti Mehra[1], Shabaana A. Khader[2], Dhiraj K. Singh[1], Deepak Kaushal[1]

Affiliations: [1]Texas Biomedical Research Institute, San Antonio, Texas, USA; [2] Department of Microbiology, University of Chicago, Chicago, Illinois, USA; [3]Houston Methodist Research Institute, Houston, Texas, USA

This PDF file includes:

Supplementary Figures and legends:

Figure S1
Figure S2
Figure S3
Figure S4
Figure S5
Figure S6
Figure S7
Figure S8

Supplementary Tables and legends:

Supplementary Table 1
Supplementary Table 2
Supplementary Table 3
Supplementary Table 4
Supplementary Table 5

Supplementary figures

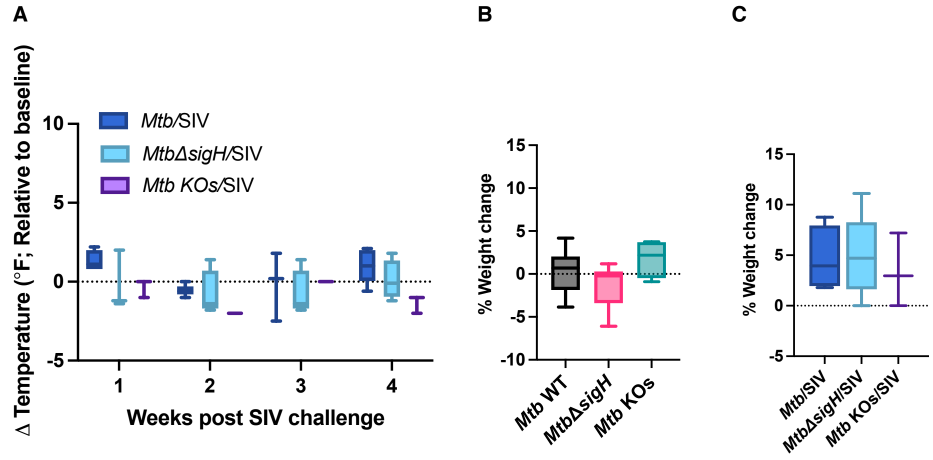

Supplementary Figure S1. Temperature change in °F **(A)** and percentage change in body weight **(C)** in *Mtb*/SIV-, *MtbΔsigH*/SIV- and *Mtb* KOs/SIV co-challenged macaques. **(B)** Percentage change in body weight of *Mtb* WT, *MtbΔsigH*- and *Mtb* KOs-challenged macaques. Data are represented as Mean <u>+</u> SEM.

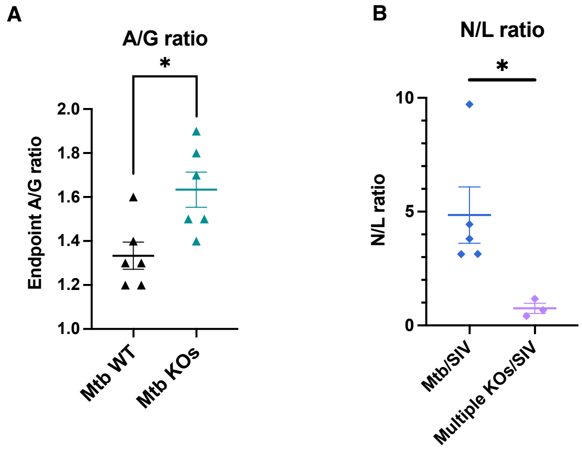

Supplementary Figure S2. **(A)** Endpoint serum Albumin/Globulin (A/G) ratios at 4 weeks post *Mtb* KOs challenge compared to those from *Mtb* WT infected macaques (**B)** blood Neutrophil (N)/Lymphocyte (L) ratios neutrophil/lymphocyte (N/L) at 4 weeks post Mtb KOs/SIV challenge compared to the ratios from *Mtb*/SIV control animals. Significance

was determined using Student's *t* test. A *P* value of <0.05 was considered as statistically significant. **P* < 0.05; ***P* <0.01; ****P* < 0.001; *****P* < 0.0001. Data are represented as Mean ± SEM.

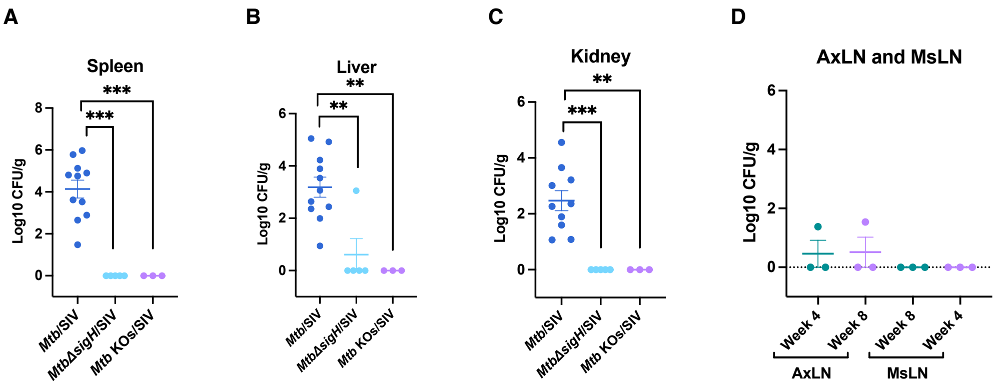

Supplementary Figure S3. Bacterial burden was determined in the spleen **(A)**, liver (**B**), kidney (**C**) and axillary (AxLN) and mesenteric (MsLN) lymph nodes (**D**) at necropsy by homogenizing the tissues and plating on agar plates. Significance was determined using one-way ANOVA with Tukey's multiple-comparisons test in GraphPad Prism v9.2.0. A *P* value of <0.05 was considered as statistically significant. **P* < 0.05; ***P* <0.01; ****P* < 0.001; *****P* < 0.0001. The data shown are the Mean ± SEM (*n* = 3).

A

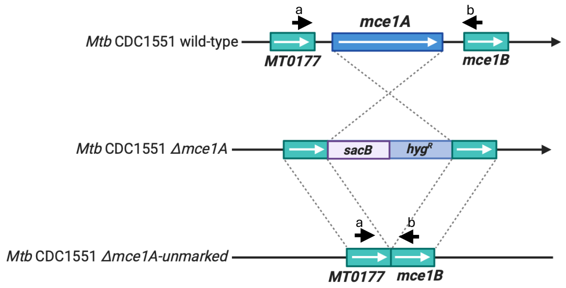

B

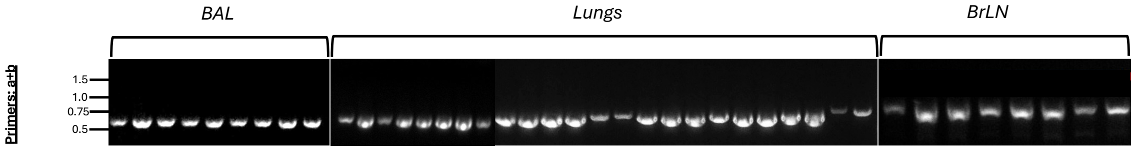

Supplementary Figure S4. (A) Schematic representation of the *mce1A* locus in the wild-type and *mce1A* unmarked mutant strain of *M. tuberculosis* (created with BioRender.com). The *mce1A* gene is approximately 1.6 kb in size. **(B)** PCR-based screening of colonies obtained from BAL, lungs and bronchial lymph nodes (BrLN) at necropsy confirming the dominating replicating bacterial strain as *MtbΔsigHΔmce1A*. In the case of an intact *mce1A*, a 1.6 kb PCR product would be expected. However, as shown here, a 575 bp amplicon was observed, indicating that all of these colonies carry the *mce1A* deletion.

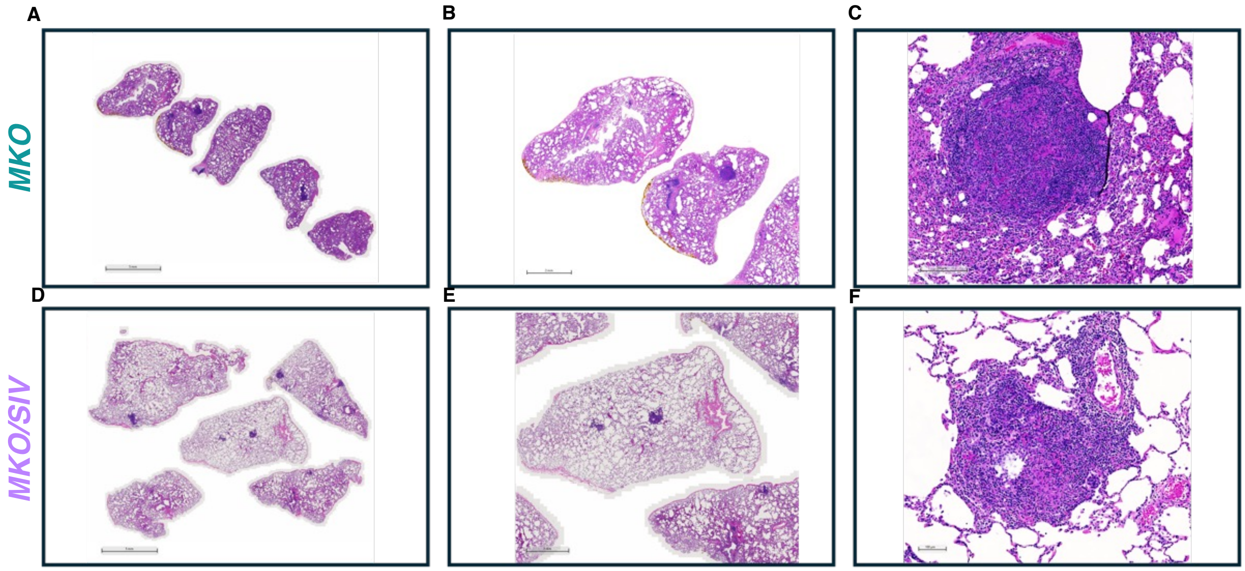

Supplementary Figure S5. Representative H&E-stained lung sections from *Mtb* KO- and *Mtb* KOs/SIV-challenged animals, indicating minimal or no gross pathology. Scale bars: 5 mm **(A and D)**, 2 mm **(B and E)**, 200 µm **(C)** and 100 µm **(F)**.

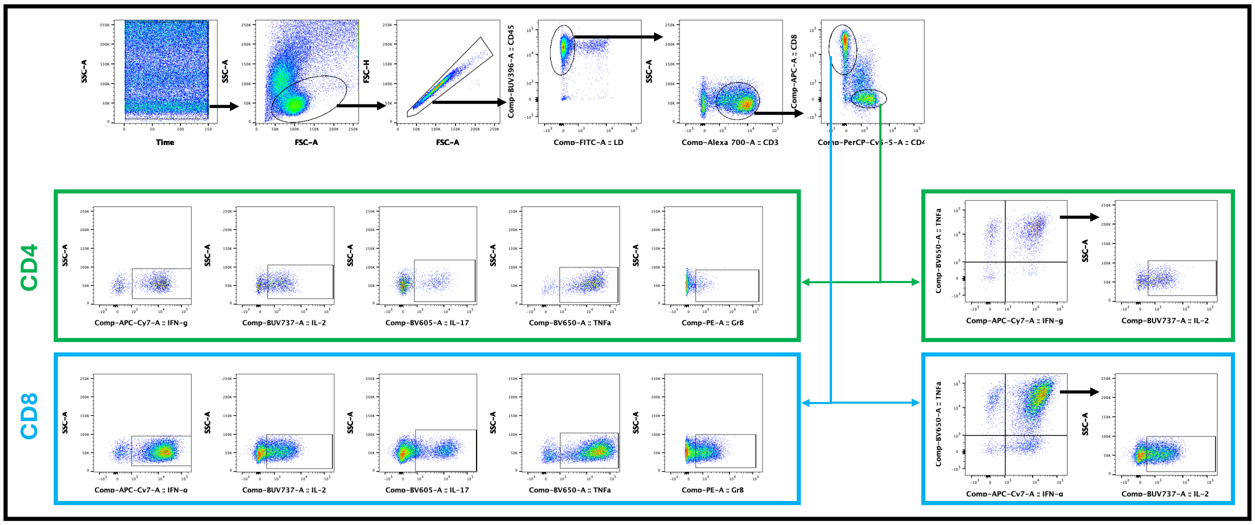

Supplementary Figure S6. Gating strategy for T cell phenotyping.

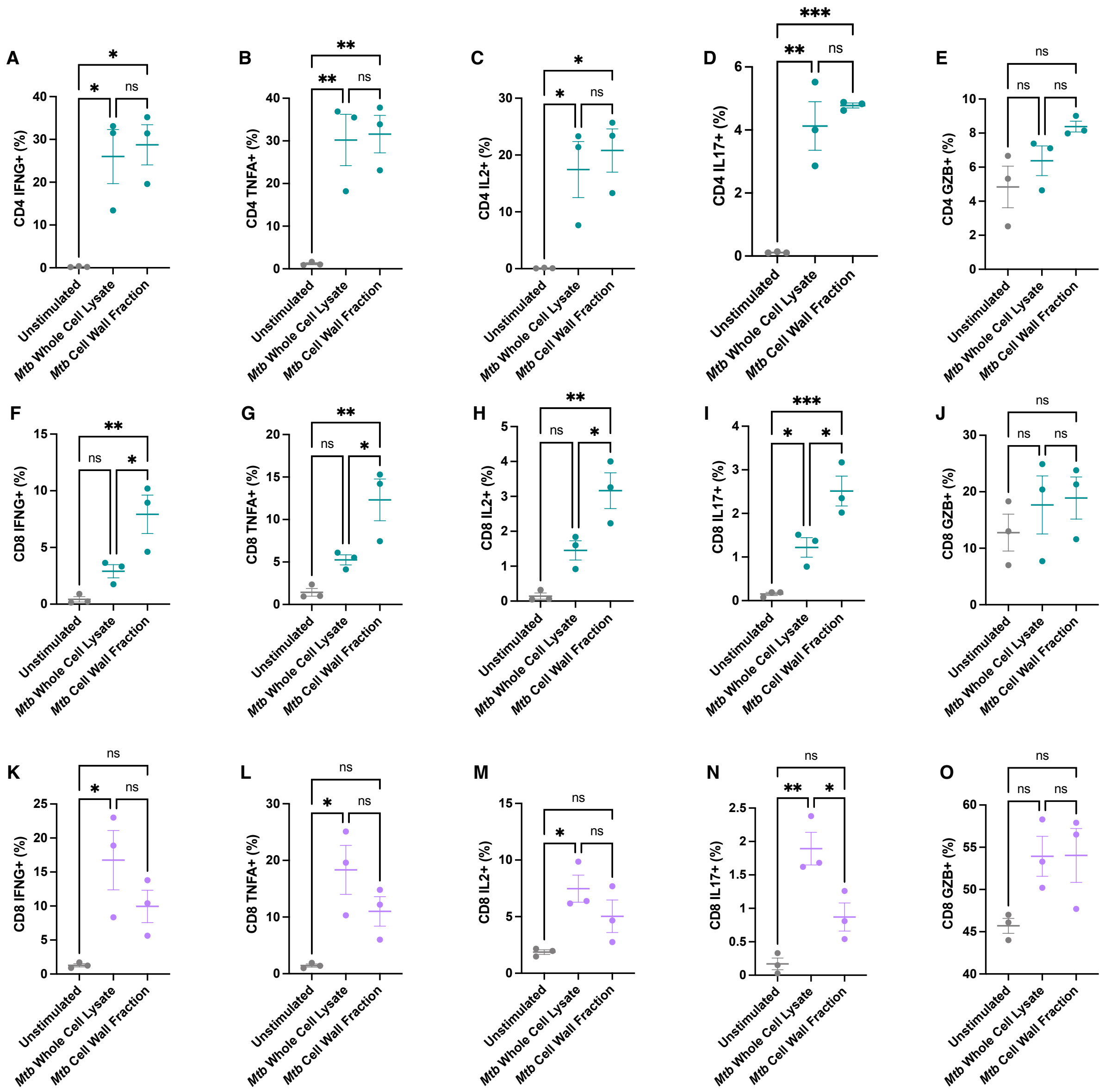

Supplementary Figure S7. Antigen specific immune responses in airways. Frequencies of antigen-specific CD4^{+} T cells expressing IFN-γ **(A)**, TNF-α **(B)**, IL-2 **(C)**, IL-17 **(D)**, GZB **(E)** and CD8^{+} T cells expressing IFN-γ **(F)**, TNF-α **(G)**, IL-2 **(H)**, IL-17 **(I)**, GZB **(J)** in BAL collected at 4 weeks post *Mtb* KOs challenge are shown. Frequencies of antigen-specific CD8^{+} T cells expressing IFN-γ **(K)**, TNF-α **(L)**, IL-2 **(M)**, IL-17 **(N)** and GZB **(O)** in BAL collected at 8 weeks post *Mtb* KOs challenge (4 weeks post SIV challenge) are shown.

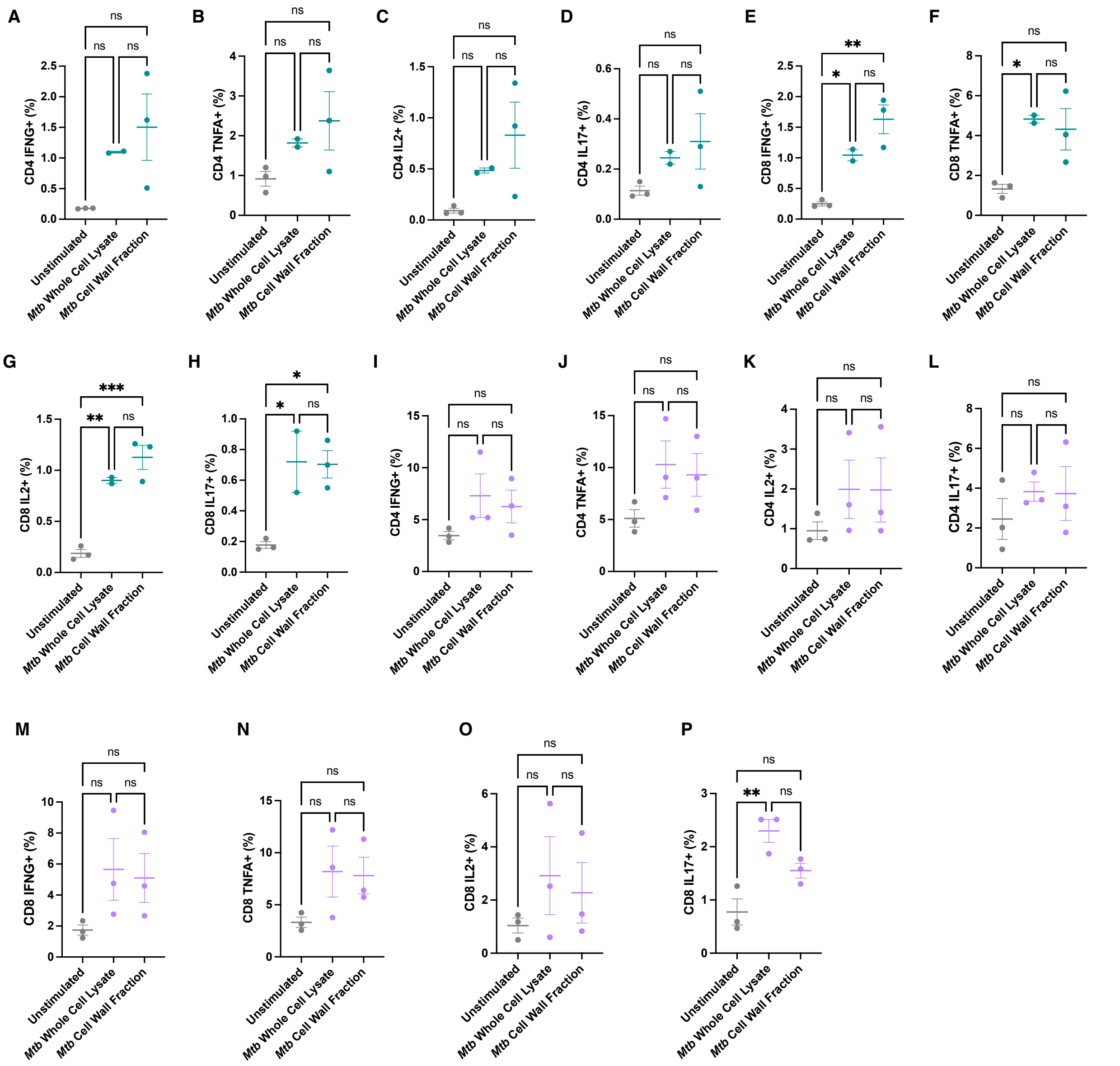

Supplementary Figure S8. Antigen specific immune responses in lungs. Frequencies of antigen-specific CD4⁺ T cells expressing IFN-γ **(A)**, TNF-α **(B)**, IL-2 **(C)**, IL-17 **(D)** and CD8⁺ T cells expressing IFN-γ **(E)**, TNF-α **(F)**, IL-2 **(G)**, IL-17 **(H)** in lung cells collected at 4 weeks post *Mtb* KOs challenge are shown. Frequencies of antigen-specific CD4⁺ T cells expressing IFN-γ **(I)**, TNF-α **(J)**, IL-2 **(K)**, IL-17 **(L)** and CD8⁺ T cells expressing IFN-γ **(M)**, TNF-α **(N)**, IL-2 **(O)**, IL-17 **(P)** in lung cells collected at 8 weeks post *Mtb* KOs challenge (4 weeks post SIV challenge) are shown.

Supplementary tables

Strain	Background	Genotype	Susceptibility to Anti-TB drugs*
DKO1	CDC1551	*ΔsigH::apra*^{r} *ΔsecA2::unmarked*	+++
DKO2	CDC1551	*ΔsigH::apra*^{r} *Δmce1A::unmarked*	+++
DKO3	CDC1551	*ΔsigH::apra*^{r} *ΔhadC::hyg*^{r}	+++
DKO4	CDC1551	*ΔsigH::apra*^{r} *Δ3785::hyg*^{r}	+++
DKO5	CDC1551	*ΔsigH::apra*^{r} *ΔleuD::hyg*^{r}	Not done
DKO6	CDC1551	*ΔsigH::apra*^{r} *ΔmetA::hyg*^{r}	Not done
TKO1	CDC1551	*ΔsigH::apra*^{r} *ΔfbpA::kan*^{r} *ΔsapM::hyg*^{r}	+++
TKO2	CDC1551	*ΔsigH::apra*^{r} *Δmce4E-mce4F::hyg*^{r}	+++

Supplementary Table 1: *ΔsigH* based M. tuberculosis deletion strains

Gene	Primer name	Forward Primer 5'-> 3'	Reverse Primer 5'-> 3'
metA	Upstream *Stu* I-*Xba* I	GGGAGGCCTATCGCCACGCCATACCTGA	GGGTCTAGACGTCTGGGTGGGTACATCGG
	Downstream *Hind* III-*Spe* I	GGGAAGCTTGCTGATCGTGAAGGCGCGTG	GGGACTAGTGGCGGGCCTGATTTACACCG
	HR	CGACCTGGAGCTTGGCTTTG	GCTCGTAGGCGCCGACCGCGG
leuD	Upstream *Stu* I-*Xba* I	GGGAGGCCTTCTGCAACATGAGCATCGAAG	GGGTCTAGAAATACCAGAGTGGGTGTGA
	Downstream *Hind* III-*Spe* I	GGGAAGCTTTGGAAACCGCGCACTCTACC	GGGACTAGTGGTCGCCGCCTTACGCACCG
	HR	GCGACGTCCAACCGCAACTTCG	CTGATTTCCAAGAGCCACGCGC
sapM	Upstream *Stu* I-*Xba* I	GGGAGGCCTACCTCGTTAAGCCCTTCGTC	GGGTCTAGAGGGCCGGCTGAGAGCCTGGA
	Downstream *Hind* III-*Spe* I	GGGAAGCTT CCGCCAATAACCGATATTTGG	GGGACTAGTGTGTCCTGCGTGGCCTCCAC
	HR	CAGCGCTGCCCGGAAGCCGG	GGTGTCGGTGCGCACGAACGC
fbpA	Upstream *Stu* I-*Xba* I	GGGAGGCCTCGTCGGGCAACTACAACCAG	GGGTCTAGACTCAACGCATCCATGCATG
	Downstream *Hind* III-*Spe* I	GGGAAGCTTAACACCGGGCCCGCGCCCC	GGGACTAGTTCCGTTGGTGTCCACACCGC
	HR	CGGCGGTGCGCTTGATGCGGGTG	GCCCGCGGCGGGAGCTAGGGAC
hadC	Upstream *Stu* I-*Xba* I	GGGAGGCCTGGACGGCGCCGCCGAACTCG	GGGTCTAGACATCCCGCGGATATCGGTCT
	Downstream *Hind* III-*Spe* I	GGGAAGCTTGAATCCGGGCAGGTCATCAG	GGGACTAGTATTACCTTCCGCATCTCGGC
	HR	CCTGAGAGCAAGTCGGTGACC	AAACCCCGAGGTCCGAGTG
MT3785	Upstream *Stu* I-*Xba* I	GGGAGGCCTGGGCTGGCGAACACCCTCGC	GGGTCTAGAGCGGATCAAGGTGGGCAAGA
	Downstream *Hind* III-*Spe* I	GGGAAGCTTCGCTCACAGCCGACAGTGTC	GGGACTAGTCCGGCCGATGCCCTCGATAC
	HR	CCCTCCCAATCGGCCTCGTG	CGTCCGCCTCGATCAACCGG
mce4E	Upstream *Stu* I-*Xba* I	GGGAGGCCTCTGGGGGATTCGCGCGGCGA	GGGTCTAGATATGATGGCGCGCAACCAGA
	Downstream *Hind* III-*Spe* I	GGGAAGCTTATTCCACCCGGTACGGCGTC	GGGACTAGTTGGCCTCGTCGATCGCATCC
mce4F	Upstream *Stu* I-*Xba* I	GGGAGGCCTCGGCTCGAGAATCACCGAGG	GGGTCTAGACAACTGGATCTTGGCGAGTC
	Downstream *Hind* III-*Spe* I	GGGAAGCTTGATCTCATGCGCGATCCGAG	GGGACTAGTTGGCGACCATCAGCGCGTA
mce4E-4F	HR	GCCGGCGCCGGGGGCAGGAC	GCGGGGCGGGACGCCTTACG
sigH	Upstream *Stu* I-*Xba* I	GGGAGGCCTGTAGGCCGCGTCGGCGTATA	GGGTCTAGACCAGCCACGTGGCTGCAGG
	Downstream *Hind* III-*Spe* I	GGGAAGCTTGAGCAGGCGCACGAGGGGGT[illegible]	GGGACTAGTCTCGGCACTTGGTCCCGATC
secA2	HR	GACCCAAGCGTGGCTTATCC	CTGCCCGAAATTTCGCAGTGG
mce1A	HR	CGAACGGCACCTACGAGCAT	CACCACCGAGACGATGCCGAG

Supplementary Table 2: Sequences of oligonucleotides

Subject	Species	Common name	Gender	Age (Years)	Weight (kg)	Group	TST Status	SIV Challenge (weeks post MKO challenge)	Necropsy (weeks post MKO challenge)
GG01	*Macaca mulatta*	Rhesus macaques	M	3	6.80	MKO	Negative	N/A	4
GG02	*Macaca mulatta*	Rhesus macaques	F	4	7.70	MKO	Negative	N/A	4
GG03	*Macaca mulatta*	Rhesus macaques	F	4	7.50	MKO	Negative	N/A	4
GG04	*Macaca mulatta*	Rhesus macaques	M	4	7.60	MKO/SIV	Negative	4	8
GG05	*Macaca mulatta*	Rhesus macaques	F	4	5.70	MKO/SIV	Negative	4	8
GG06	*Macaca mulatta*	Rhesus macaques	M	4	8.90	MKO/SIV	Negative	4	8

Supplementary Table 3: Demographics for individual animals used in the study

S. No.	Marker	Flourochrome	Clone
1	CD45	BUV395	D058-1283
2	IL-2	BUV737	MQ1-17H12
3	CD95	BV421	DX2
4	IL-17	BV605	BL168
5	TNF-a	BV650	Mab11
6	Live/Dead	FITC	
7	CD4	PerCP-Cy5.5	L200
8	Granzyme B	PE	GB11
9	CD28	PE-Cy7	CD28.2
10	CD8	APC	RPA-T8
11	CD3	AL700	SP34-2
12	IFN-g	APC-CY7	B27

Supplementary Table 4: Flow Cytometry panel for measuring antigen specific T cell responses.

Antibody	Supplier	Clone	Cat Number	Validation Statement: Reactivity	Dilution
CD45 (BUV395)	BD Bioscience	D058-1283	564099	Rhesus, Cynomolgus, Baboon (QC Testing)	As recommended by Manufacturer
IL-2 (BUV737)	BD Bioscience	MQ1-17H12	612836	Human (QC Testing) Rhesus, Cynomolgus, Baboon (Tested in Development)	As recommended by Manufacturer
CD95 (BV 421)	BD Bioscience	DX2	562616	Human (QC Testing) Rhesus, Cynomolgus, Baboon (Tested in Development)	As recommended by Manufacturer
IL-17 (BV605)	Biolegend	BL168	512326	Human, Rhesus (Validated in lab, Reported)	As recommended by Manufacturer
TNF-alpha (BV650)	Biolegend	MAb11	502938	Human, Cat (Feline)11 Cross-Reactivity: Chimpanzee, Baboon, Cynomolgus, Rhesus, Pigtailed Macaque, Sooty Mangabey, Swine (Pig, Porcine)	As recommended by Manufacturer
CD4 (PCP-Cy5.5)	BD Bioscience	L200	552838	Rhesus, Cynomolgus, Baboon (QC Testing) Human (Tested in Development)	As recommended by Manufacturer
GrB (PE)	BD Bioscience	GB11	561142	Human (QC Testing), Rhesus (NHP Reagent Resource)	As recommended by Manufacturer
CD28 (PE-Cy7)	BD Bioscience	CD28.2	560684	Human (QC Testing), Rhesus (NHP Reagent Resource)	As recommended by Manufacturer
CD8 (APC)	Biolegend	RPA-T8	301049	Chimpanzee, Baboon, Cynomolgus, Rhesus, Pigtailed Macaque, Sooty Mangabey	As recommended by Manufacturer
CD3 (AL700)	BD Bioscience	SP34-2	557917	Rhesus, Cynomolgus, Baboon (QC Testing) Human (Tested in Development)	As recommended by Manufacturer
IFN-G (APC-Cy7)	Biolegend	B27	506524	Chimpanzee, Baboon, Cynomolgus, Rhesus, Pigtailed Macaque, African Green, Sooty Mangabey	As recommended by Manufacturer
CD68	Roche	KP-1	790-2931	Mouse, monoclonal	As recommended by Manufacturer
CD20	Roche	L26	760-2531	Mouse, monoclonal	As recommended by Manufacturer
CD3	Roche	2GV6	790-4341	Rabbit, monoclonal	As recommended by Manufacturer

Supplementary Table 5: List of antibodies.
