# Supplementary material for: Development and preclinical evaluation of next-generation ΔsigH-based live candidate vaccines
Source: JCI Insight. 2025 Aug 28;10(19):e195947. doi: 10.1172/jci.insight.195947 (PMC12513488; doi:10.1172/jci.insight.195947)

**Full unedited gel for Figure 1B (Lanes 10 and 12), 1F (Lanes 3 and 4), 1N (Lanes 23 and 24), 1O (Lanes 6 and 8) and 1Q (Lanes 19 and 20)**

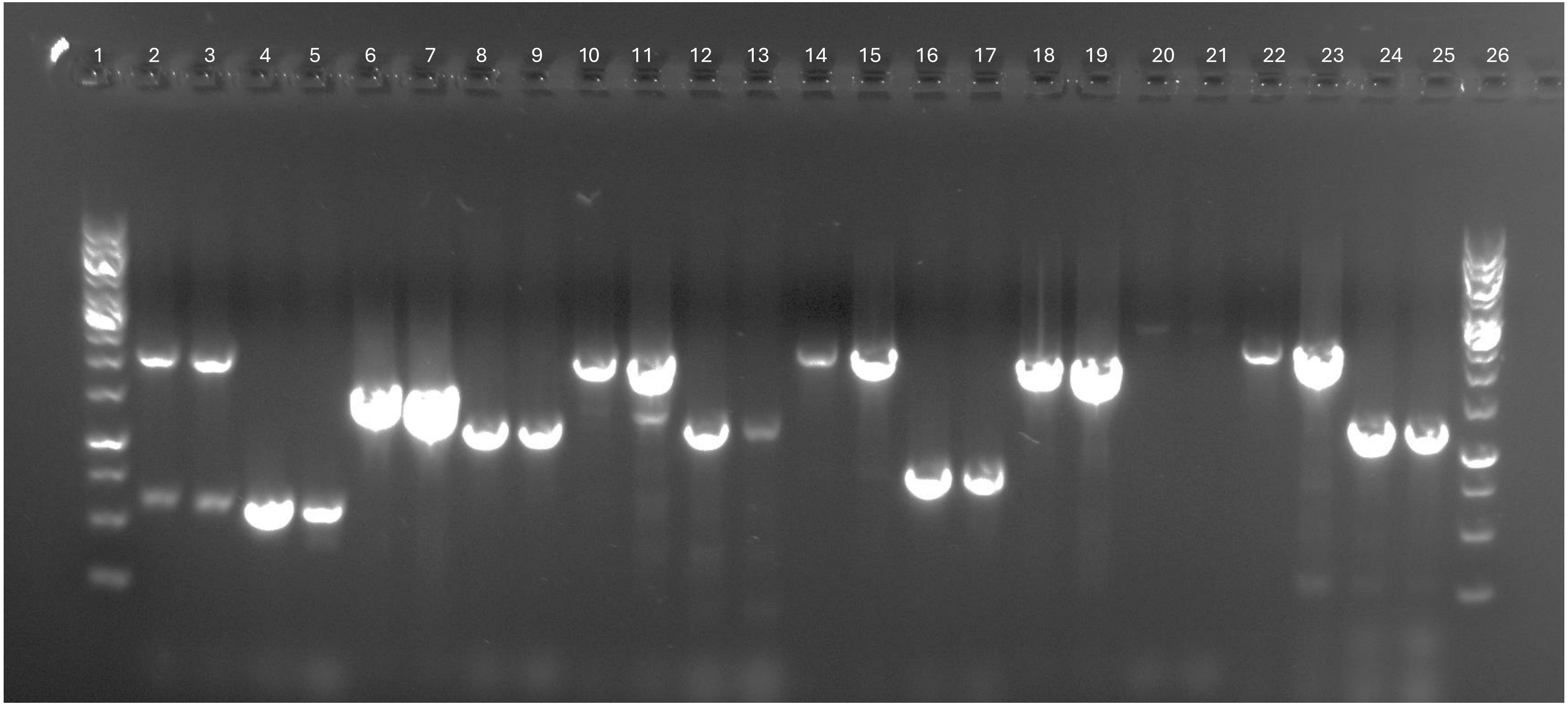

## Full unedited gel for Figure 1 D (Lanes 6 and 7) and 1H (Lanes 16 and 17)

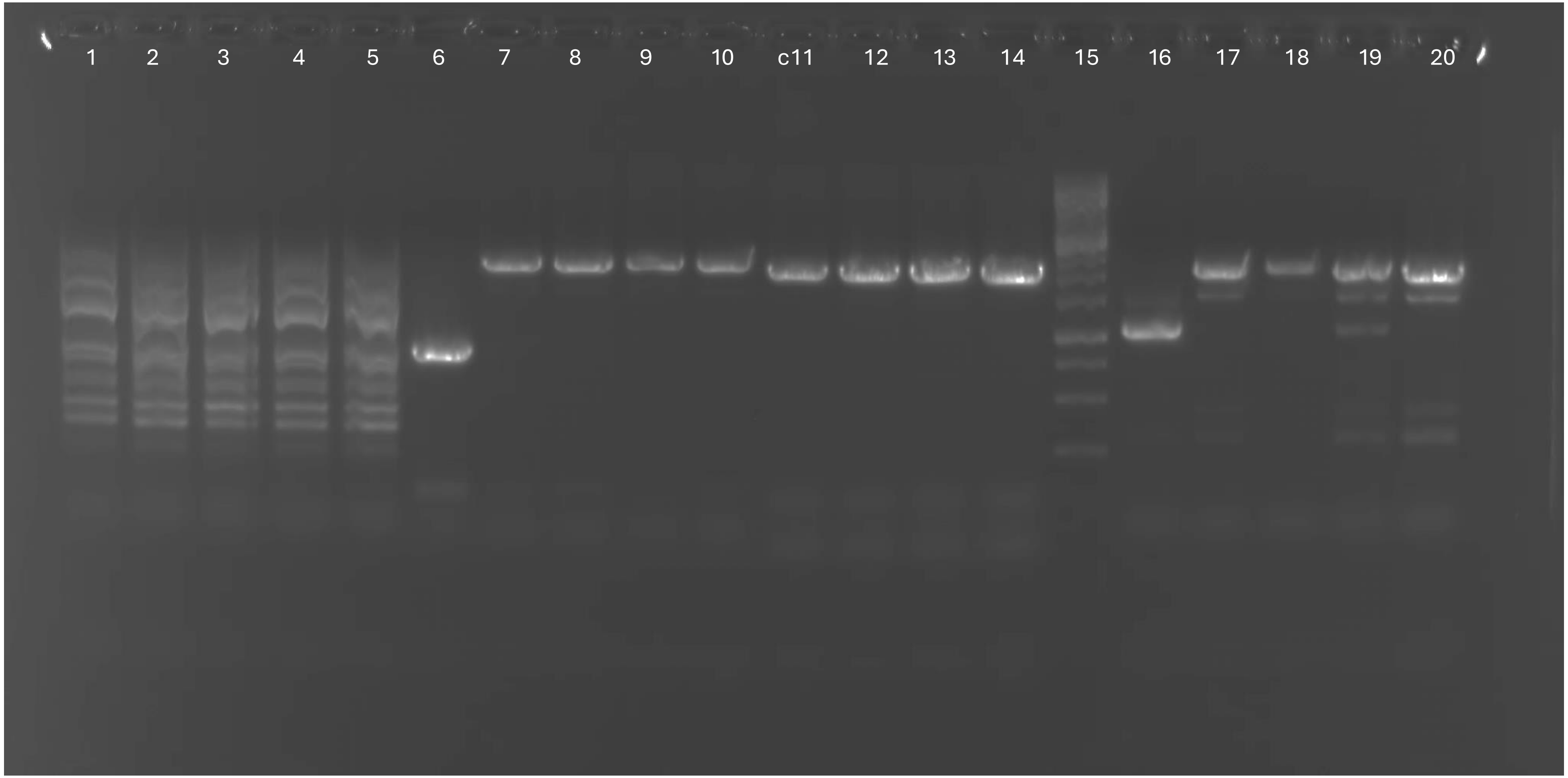

**Full unedited gel for Figure 1 J (Lanes 16 and 17)**

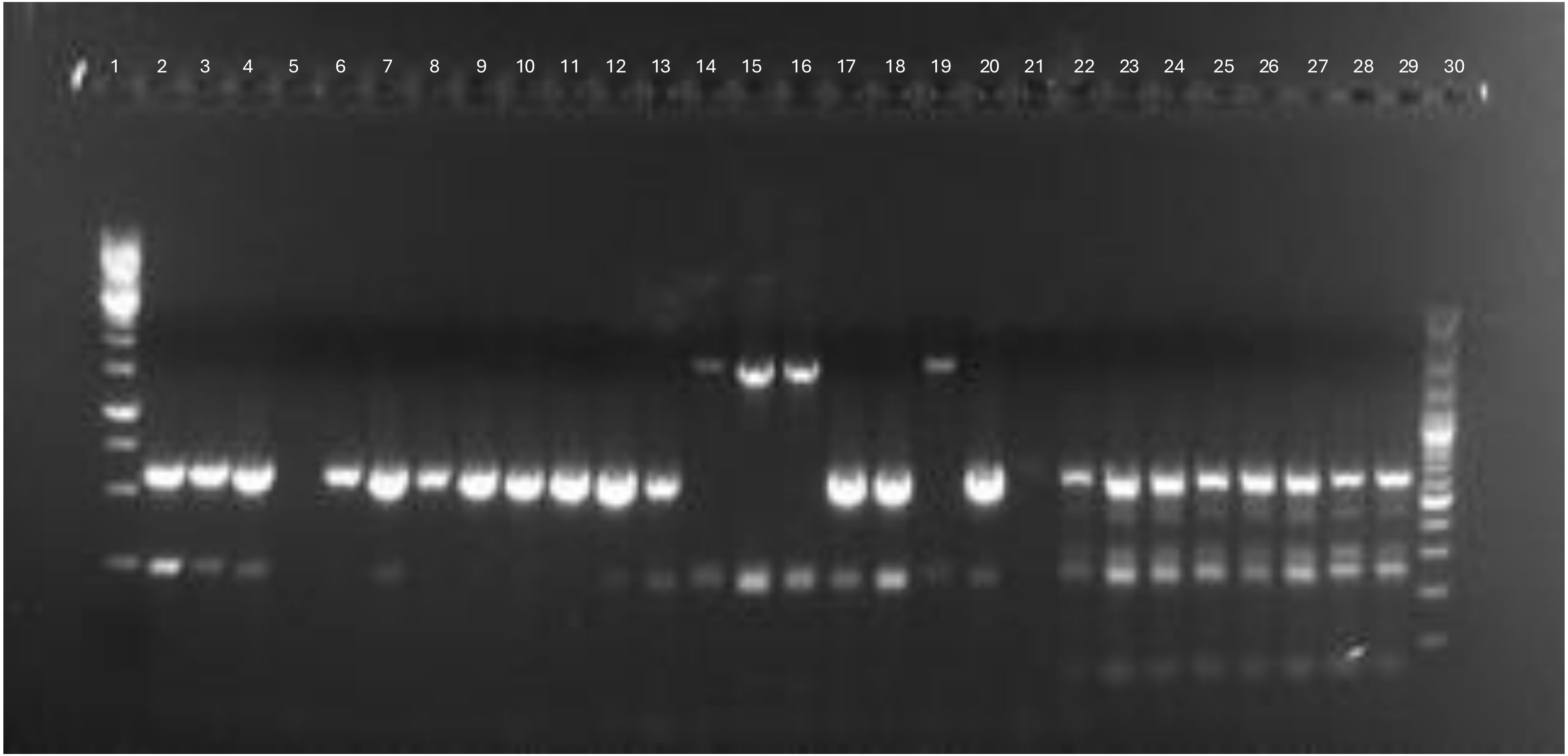

# Full unedited gel for Figure 1 L (Bottom lanes 8 and 9)

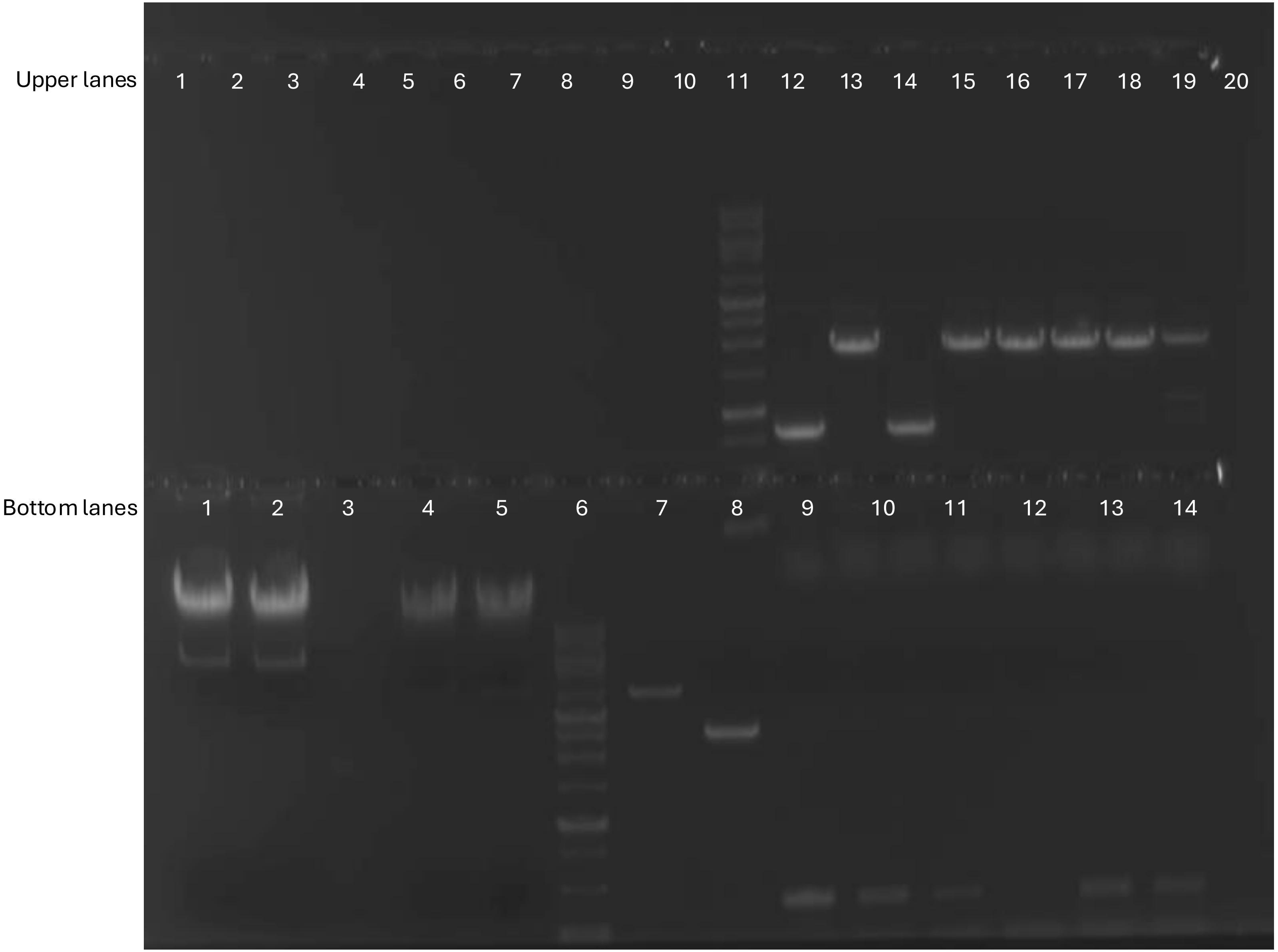

# Full unedited gel for Figure 1S (Bottom lanes 1 and 2)

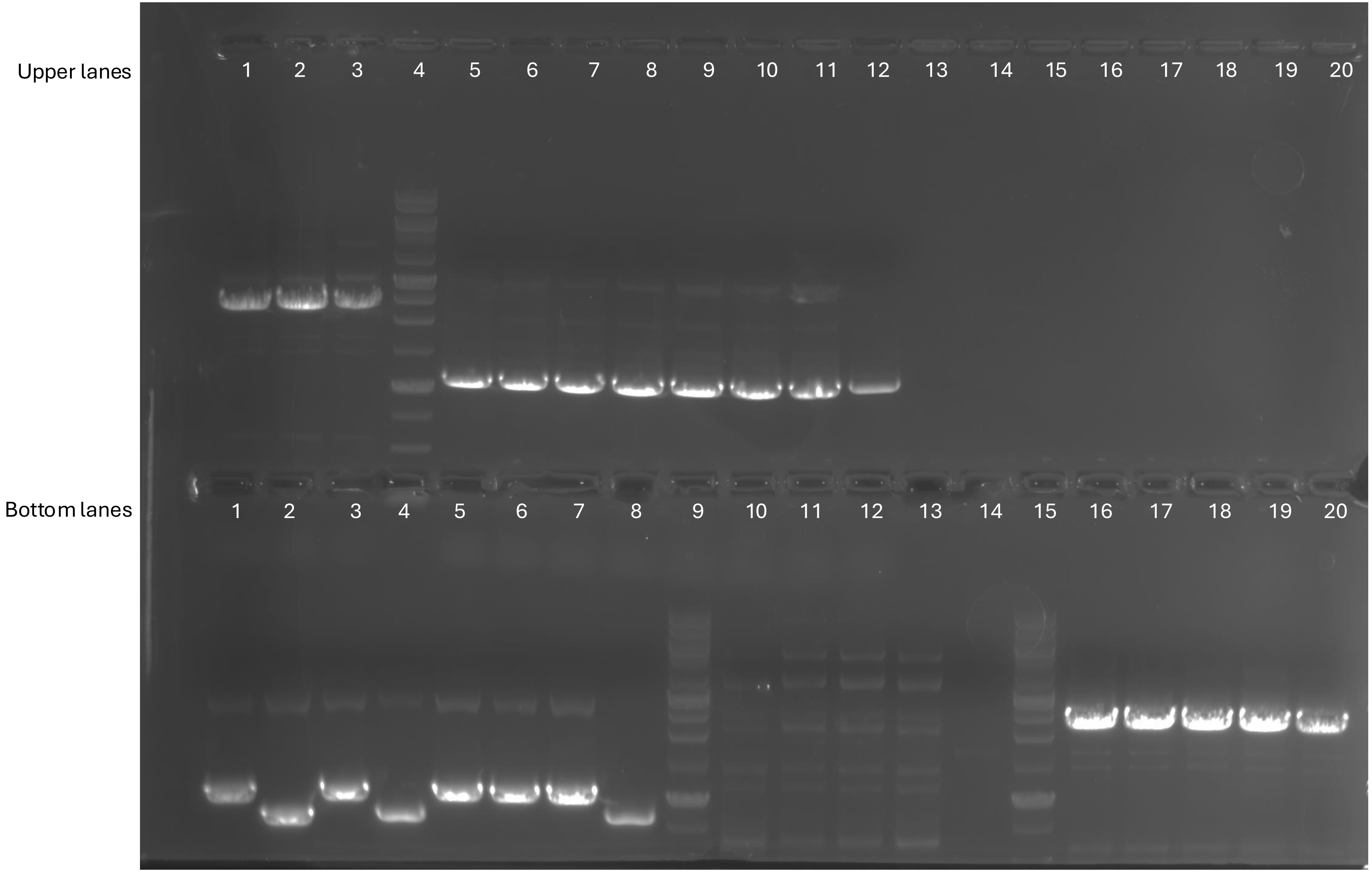

# Full unedited gel for Supplementary Figure S4B – BAL (Lanes 6-14)

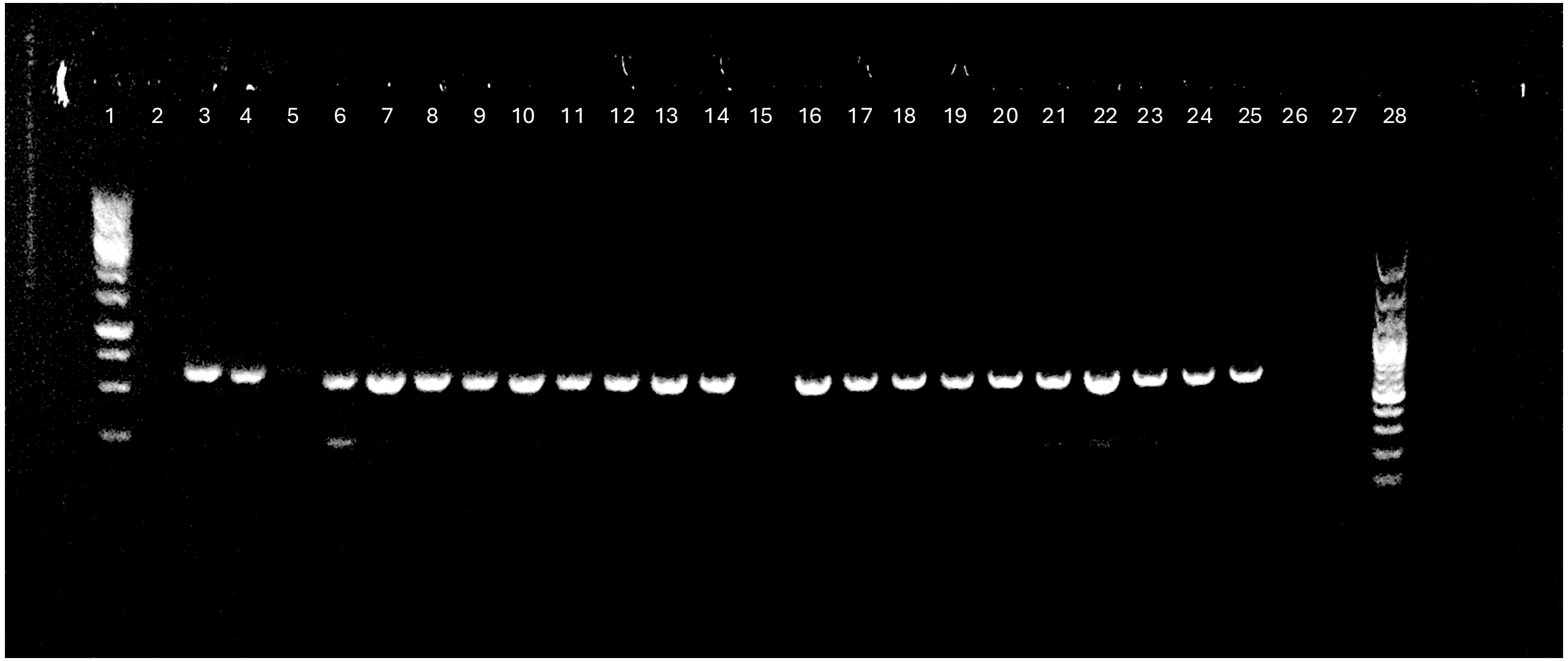

**Full unedited gels for Supplementary Figure S4B – Lungs (Lanes 6-14 in Gel 1 and Lanes 1-25 in Gel 2)**

Gel 1

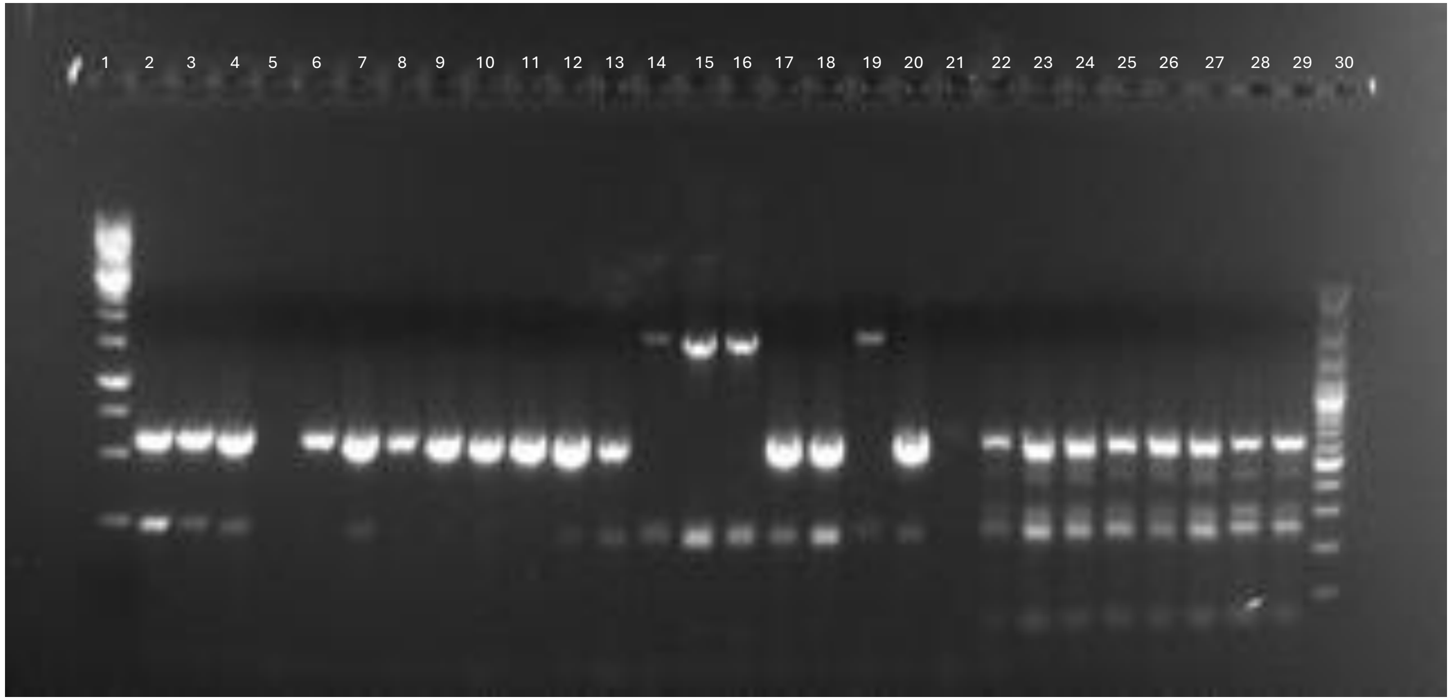


Gel 2

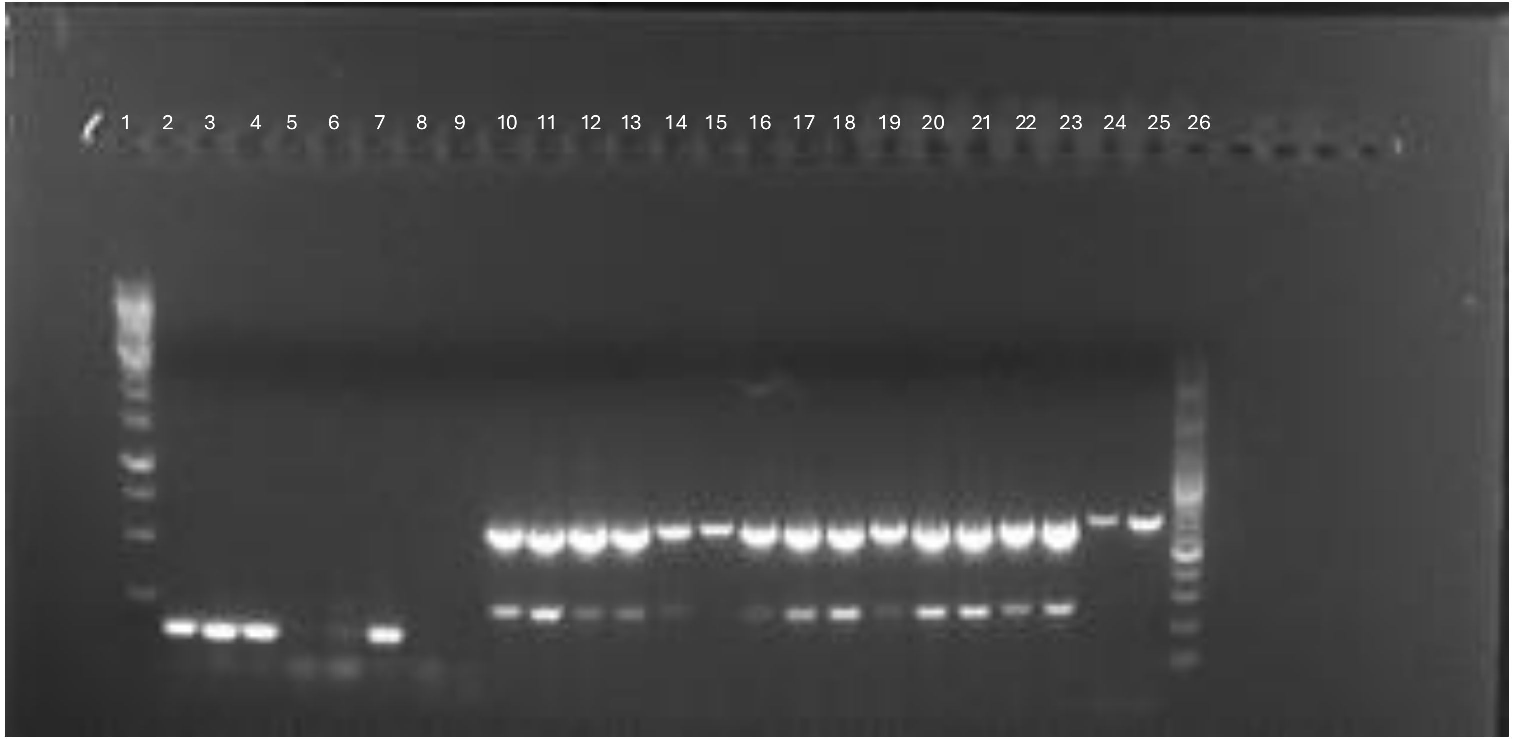

## Full unedited gel for Supplementary Figure S4B – BrLN (Lanes 23-29)

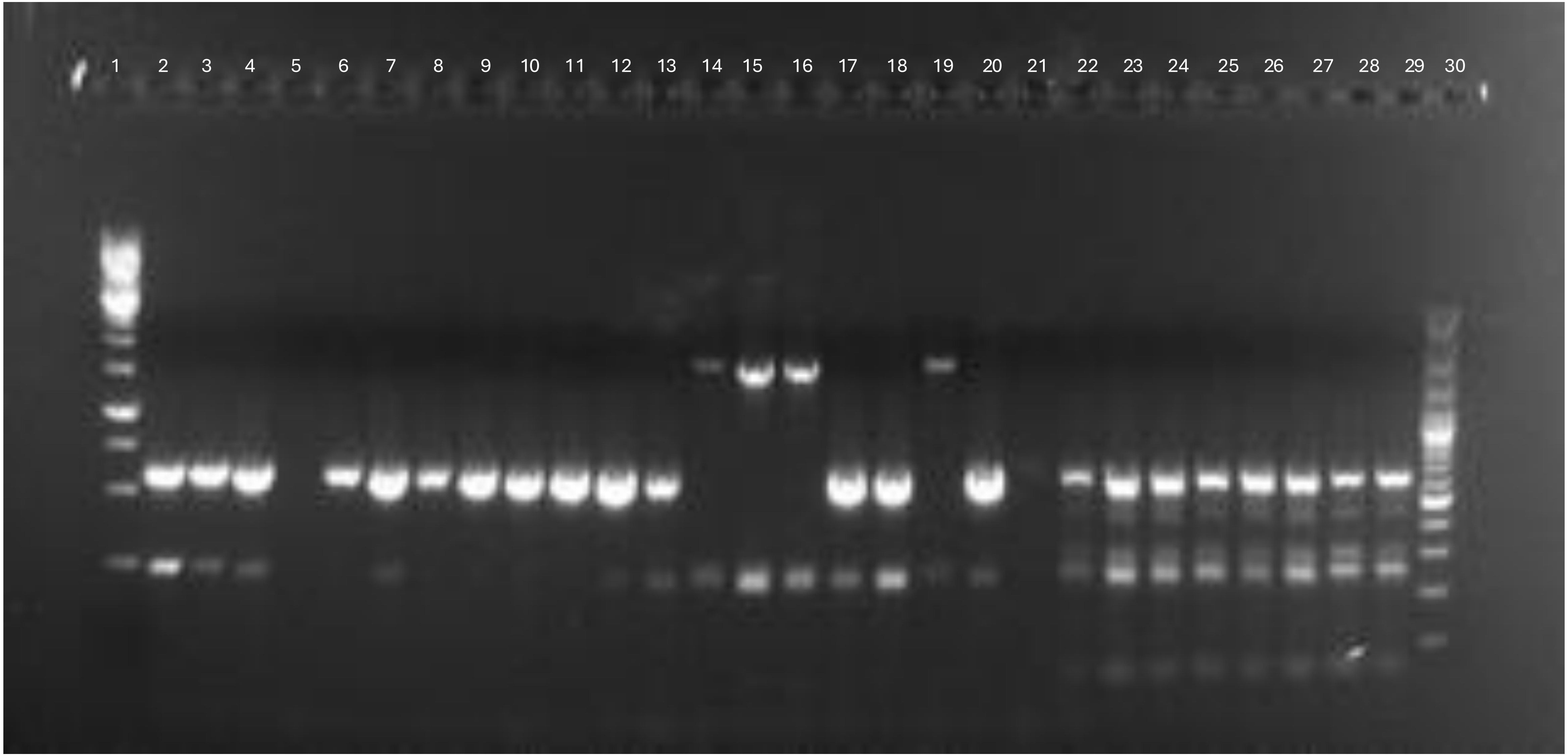

Supplement: Unedited blot and gel images [file jciinsight-10-195947-s238.pdf]
